# An early‐senescence state in aged mesenchymal stromal cells contributes to hematopoietic stem and progenitor cell clonogenic impairment through the activation of a pro‐inflammatory program

**DOI:** 10.1111/acel.12933

**Published:** 2019-03-03

**Authors:** Daniela Gnani, Stefania Crippa, Lucrezia della Volpe, Valeria Rossella, Anastasia Conti, Emanuele Lettera, Silvia Rivis, Marco Ometti, Gianfranco Fraschini, Maria Ester Bernardo, Raffaella Di Micco

**Affiliations:** ^1^ San Raffaele Telethon Institute for Gene Therapy Milan Italy; ^2^ Vita‐Salute San Raffaele University Milan Italy; ^3^ Department of Orthopedics and Traumatology San Raffaele Hospital Scientific Institute Milan Italy; ^4^ Pediatric Immunohematology and Bone Marrow Transplantation Unit San Raffaele Scientific Institute Milan Italy; ^5^ Present address: Swiss Stem Cell Biotech Vacallo Switzelard

**Keywords:** aging, DNA damage, hematopoietic stem and progenitor cells, inflammation, mesenchymal stromal cells, SASP, senescence

## Abstract

Hematopoietic stem and progenitor cells (HSPC) reside in the bone marrow (BM) niche and serve as a reservoir for mature blood cells throughout life. Aging in the BM is characterized by low‐grade chronic inflammation that could contribute to the reduced functionality of aged HSPC. Mesenchymal stromal cells (MSC) in the BM support HSPC self‐renewal. However, changes in MSC function with age and the crosstalk between MSC and HSPC remain understudied. Here, we conducted an extensive characterization of senescence features in BM‐derived MSC from young and aged healthy donors. Aged MSC displayed an enlarged senescent‐like morphology, a delayed clonogenic potential and reduced proliferation ability when compared to younger counterparts. Of note, the observed proliferation delay was associated with increased levels of SA‐β‐galactosidase (SA‐β‐Gal) and lipofuscin in aged MSC at early passages and a modest but consistent accumulation of physical DNA damage and DNA damage response (DDR) activation. Consistent with the establishment of a senescence‐like state in aged MSC, we detected an increase in pro‐inflammatory senescence‐associated secretory phenotype (SASP) factors, both at the transcript and protein levels. Conversely, the immunomodulatory properties of aged MSC were significantly reduced. Importantly, exposure of young HSPC to factors secreted by aged MSC induced pro‐inflammatory genes in HSPC and impaired HSPC clonogenic potential in a SASP‐dependent manner. Altogether, our results reveal that BM‐derived MSC from aged healthy donors display features of senescence and that, during aging, MSC‐associated secretomes contribute to activate an inflammatory transcriptional program in HSPC that may ultimately impair their functionality.

## 1 INTRODUCTION

Hematopoietic stem and progenitor cells (HSPC) can self‐renew and differentiate into all blood components thus serving as a reservoir for mature blood cells throughout life. However, as we age, HSPC functionality is impaired with cells displaying a reduced capacity to maintain tissue homeostasis (Geiger, de Haan, & Florian, 2013). Indeed, although the number of phenotypically defined HSPC increases with aging, aged HSPC display impaired regenerative capacity when transplanted into host recipients and a differentiation skewing toward the myeloid hematopoietic lineage (Geiger et al., 2013).

Hematopoietic stem and progenitor cells reside in the BM niche, and their function is supported by a variety of both hematopoietic and nonhematopoietic cell types, such as osteoblasts, adipocytes, endothelial, and mesenchymal stromal cells (MSC) (Morrison & Scadden, 2014). Among these, BM‐derived MSC are multipotent cells of mesodermal origin capable of adhering to culture dishes, proliferate in vitro, and differentiate into different lineages, including osteoblasts, adipocytes, and chondrocytes. Several studies highlighted the key role of MSC in regulating HSPC fate and promoting engraftment of the rare and more primitive hematopoietic stem cells (HSC) (Kfoury & Scadden, 2015). Even if the molecular mechanisms that regulate HSPC dysfunction in the elderly are thought to be primarily cell‐intrinsic, recent insights support the contribution of external cues from the niche to HSPC dysfunction during aging (Adams et al., 2007; Geiger et al., 2013; Mendez‐Ferrer et al., 2010). Indeed, changes in the cellular composition of the HSC niche during aging contribute to hematologic decline and involve decreased bone formation, enhanced adipogenesis, increased BM inflammation, and altered HSPC‐MSC crosstalk (Mendelson & Frenette, 2014). Consistent with this, some studies, mainly conducted in mouse settings, have reported that MSC decrease in number with aging and display a more pronounced differentiation toward the adipogenic lineage at the expenses of bone formation (Liu, Xia, & Li, 2015).

Senescent cells accumulate during aging (Campisi, 2005; Farr et al., 2017) and contribute to tissue dysfunction and impaired tissue regeneration. Senescent cells display an enlarged morphology coupled with a proliferation arrest mediated by the Rb/p16 and p53/p21 pathways. Senescence is also characterized by increased SA‐β‐Gal activity, persistent DDR activation, and telomeric attrition. Moreover, senescent cells exhibit transcriptional activation of a senescent‐associated secretory inflammatory phenotype collectively known as SASP (Coppe et al., 2008). The robust secretion of SASP chemokines/cytokines triggers an inflammatory response that could reinforce senescence in a cell‐autonomous fashion and be transferred to surrounding cells through paracrine mechanisms, to amplify the senescence response. To date, the activation of a senescence program in human aged MSC and the interplay between aged MSC and HSPC remain to be elucidated. In this study, we successfully established human BM‐derived MSC from young and elderly healthy donors. We investigated the effects of chronological age on MSC properties and found that MSC derived from aged healthy subjects show senescence‐like features comprising an enlarged morphology, reduced proliferation capacity, delayed cell cycle progression, and increased levels of SA‐β‐Gal and lipofuscin. Importantly, we found that aged MSC activate a SASP‐like program that contributes in a non cell autonomous manner to impair young HSPC clonogenicity by mediating an inflammatory state in HSPC.

## 2 RESULTS

### 2.1 Aged MSC display reduced clonogenic capacity compared to younger counterparts

To identify molecular determinants of MSC aging, we derived MSC from bone marrow (BM) of healthy subjects belonging to three different age groups: pediatric (<18 years) (*n* = 4), young adults (18–35 years) (*n* = 6), and aged subjects (≥70) (*n* = 12) (See Experimental Procedures section for details). Pediatric and young adult subjects were BM donors for hematopoietic stem cell transplantation (HSCT), while BM samples from aged donors were collected from healthy subjects undergoing primary hip replacement. In the efforts to identify features of healthy chronological aging in MSC and to avoid any confounding effect due to underlying age‐related pathologies, we excluded from our analysis subjects with compromised immune functions and previous history of cancer or cancer treatments, even if most elderly subjects in our cohort were diagnosed with hypertension. Of note, none of the elderly subjects under study displayed symptoms of overt frailty upon clinical assessment.

**Figure 1:**
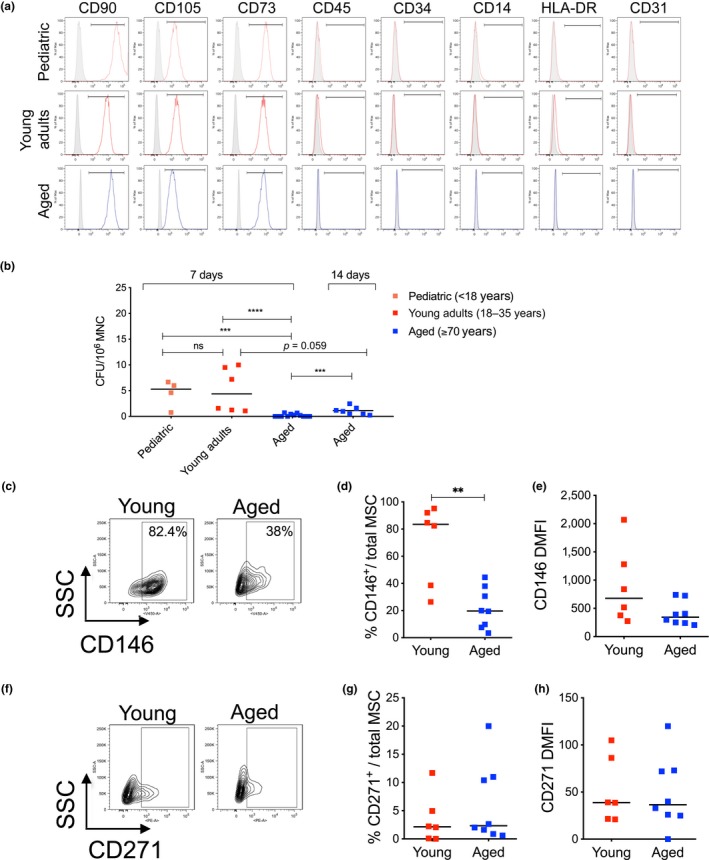
Biological characterization of young and aged MSC. (a) Representative flow cytometric plots of immunophenotypic characterization of MSC from pediatric, young adults, and aged subjects. Canonical MSC markers: CD90, CD105, and CD73. Hematopoietic markers: CD45, CD34, and CD14. MHC class II marker: HLA‐DR. Endothelial marker: CD31. (b) Colony‐formation assay (CFU‐F) at day 7 and day 14 after the initial seeding. Data are expressed as CFU/10^6^ MNC plated and shown as scatter dot plot; lines indicate median values (pediatric, *n* = 4; young adults, *n* = 6; aged, *n* = 12). *p*‐value was determined by Mann–Whitney test; ****p  < 0.0001; ***p < 0.001. (c) Representative flow cytometric analysis of CD146 in young and aged MSC. (d) Frequency of CD146‐positive cells and (e) level of CD146 expression (DMFI) in young and aged MSC (young, *n* = 6; aged, *n* = 8); *p*‐value was determined by Mann–Whitney test; **p  < 0.01. (f) Representative flow cytometric analysis of CD271 in young and aged MSC. (g) Frequency of CD271‐positive cells and (h) level of CD271 expression in young and aged MSC (young, *n* = 6; aged, *n* = 8). In all panels, each squared dot represents an individual MSC donor (red = young; blue = aged)

We isolated mononuclear cells (MNC) from BM by density gradient centrifugation; after depletion of CD34^+^ cells purified from MNC by immunomagnetic selection, we plated the remaining CD34^−^ fraction for MSC ex vivo expansion in culture medium supplemented with 5% platelet lysate, state‐of‐the‐art protocol for derivation of human MSC for clinical use in transplantation settings and regenerative medicine (Avanzini et al., 2009; Ingo et al., 2016). First, we verified that established MSC from young and aged BM conform to the International Society for Cell & Gene Therapy (ISCT) minimal definition criteria that include plastic adherence and spindle‐shape morphology, immunophenotype, and multilineage differentiation potential (Dominici et al., 2006). We first analyzed MSC samples from the three age groups by flow cytometry and evaluated the expression of established MSC surface markers. We observed that MSC from pediatric, young adults, and aged subjects expressed canonical MSC markers including CD90, CD105, and CD73 and were negative for the expression of hematopoietic/endothelial markers such as CD45, CD34, CD14, CD31, and HLA‐DR (representative examples shown in Figure 1a). We next evaluated the clonogenic potential of MSC by measuring colonies formed unit‐fibroblasts (CFU‐F), a property of the more primitive enriched MSC subsets (Sacchetti et al., 2007; Tormin et al., 2010). At the time of MSC derivation, we counted the number of clones generated at 7 and 14 days normalized for the number of MNC seeded. Interestingly, despite some individual donor variability, we did not detect any significant difference in the clonogenic capacity of MSC derived from pediatric and young adult donors (hereafter used as a unified group and defined as “young”). Instead, MSC from aged individuals displayed a significant reduction of CFU‐F ability at day 7 compared to younger counterparts that was partially rescued at day 14 (Figure 1b).

In order to identify intrinsic alterations in MSC phenotype during advanced aging and their impact on hematopoietic stem cell functionality, we randomly selected up to 8 aged MSC samples and consistently analyzed them in comparison with up to 6 MSC samples from the “young” group. To minimize the effect of culture‐induced alterations on MSC biology and functionality and better resemble MSC properties in the BM, we focused all our analyses on ex vivo expanded MSC at early passages in culture (between passage P2 and P4).

Given that the CFU‐F ability was impaired in aged MSC, we evaluated the expression of two different surface markers that are used to identify primitive MSC, CD146 (melanoma cell adhesion molecule—MCAM) (Sacchetti et al., 2007), and CD271 (nerve growth factor receptor—NGFR) (Mabuchi et al., 2013). Flow cytometric analysis revealed a significant decrease in the % of CD146^+^ cells and reduced intensity of CD146 expression in the analyzed aged MSC when compared to young MSC (Figure 1c–e), whereas the frequency and intensity of CD271^+^ MSC were not altered during aging (Figure 1f–h).

We next evaluated the differentiation potential of young and aged MSC. Early passage MSC were induced to differentiate into osteogenic and adipogenic lineages by culturing the cells for three weeks (day 21) in the presence of media enriched in dexamethasone, l‐ascorbic acid, insulin, methylxanthine, indomethacin, and β‐glycerol phosphate for adipocyte differentiation, and dexamethasone, l‐ascorbic acid, and β‐glycerol phosphate for development of the osteogenic lineage. When we measured the levels of expression of adipogenic genes (*PPARγ, FABP4, LPL*) at steady state, we did not observe any significant difference among young and aged MSC (Supporting information Figure S1a–c), except for a decrease in *PPARγ *levels in aged MSC (Supporting information Figure S1a). At day 21 of differentiation, aged MSC displayed a similar ability to differentiate toward adipocytes compared to younger counterparts as revealed by induction of *PPARγ *levels, a key player involved in the initial phase of adipocyte differentiation, and FABP4 and LPL genes, involved in the later phases of adipocytes commitment (Supporting information Figure S1d–f). Consistent with this, young and aged MSC displayed similar accumulation of lipid droplets upon differentiation induction, as shown by Oil Red O staining (Supporting information Figure S1g). We next evaluated the levels of genes involved in osteogenic differentiation (*RUNX2, SPARC, COL1A2*) and observed comparable mRNA levels in young and aged MSC at steady state (Supporting information Figure S1h–j). Upon 21 days of differentiation, we observed significant induction of osteogenic genes in young MSC, while the levels of *RUNX2, SPARC, and COL1A2 *did not reach significance in differentiated aged MSC. Moreover, we reported a decrease in the induction of *RUNX2 *and *COL1A2 *in aged MSC compared to young MSC upon differentiation (Supporting information Figure S1k–m). Accumulation of calcium deposition as indicated by alizarin red staining was similar in young and aged MSC (Supporting information Figure S1n).

Altogether, these data indicate that MSC were successfully derived from aged BM and displayed an impaired clonogenic potential associated with a significant reduction of mesenchymal primitive cell surface markers and with a mild impairment in the differentiation ability toward the osteogenic lineage.

### 2.2 An early‐senescence state characterizes aged human BM‐derived MSC

**Figure 2:**
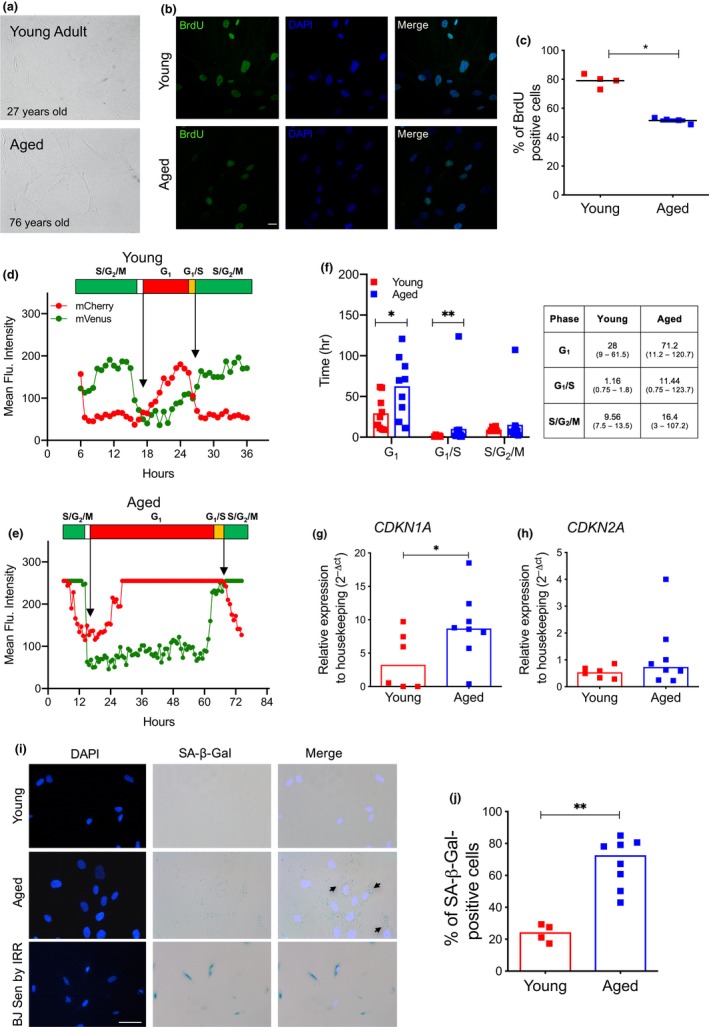
Aged MSC are characterized by an early senescent state. (a) Representative brightfield images of BM‐derived MSC isolated from young and aged donors at early passages in culture. (b) Representative confocal images of BrdU incorporation assay in young and aged MSC. DAPI indicates nuclei, scale bar = 20µm (young, *n* = 4; aged, *n* = 4). Quantification of BrdU‐positive cells is shown in (c). *p*‐value was determined by Mann–Whitney test; *p < 0.05. (d–e) Representative cell cycle kinetics of young (d) and aged (e) MSC as determined by mean fluorescent intensity from Fucci2A‐transduced cells. (f) Quantification of cell cycle kinetics of MSC from one young and two aged MSC donors measured in hours. 10 cells per each donor were analyzed in live imaging up to 6 days. Each squared dot represents a complete phase of cell cycle. Data from two aged MSC samples were represented as a unified group. The median duration of cell cycle phases, with the minimum and maximum length in brackets (hours), is reported in the table. *p*‐value was determined by Mann–Whitney test; **p  < 0.01; *p < 0.05; ns *p* > 0.05. (g–h) Relative mRNA expression of *CDKN1A *(g) and *CDKN2A *(h) as measured by quantitative Real‐Time PCR. Gene expression data are represented as 2^−△CT^ relative to *GUSB *housekeeping. Each squared dot represents an individual MSC donor (young, *n* = 6; aged, *n* = 8) (red = young; blue = aged); lines indicate median 2^−△CT^ values. *p*‐value was determined by Mann–Whitney test; *p < 0.05. (i) Representative pictures and quantification (j) of SA‐β‐Gal‐positive MSC isolated from young and aged donors at early passages. Human senescent fibroblasts (BJ) induced into senescence by irradiation are shown as positive control for SA‐β‐Gal staining in (i). DAPI was used to stain nuclei. Scale bar = 50 µm (young, *n* = 4; aged, *n* = 8). *p*‐value was determined by Mann–Whitney test; **p  < 0.01

Having observed a reduced clonogenic capacity of aged MSC compared to younger counterparts, we asked whether aged MSC display reduced proliferation capacity and induction of cell senescence markers even at early passages in culture. First, we noticed that the morphology of aged MSC was more flat and enlarged compared to spindle‐shaped young cells, resembling senescent fibroblasts (Figure 2a). Since fully senescent cells are characterized by a stable cell cycle arrest, we measured the proliferative ability of young and aged MSC. We carried out bromodeoxyuridine (BrdU) incorporation assays to evaluate the percentage of MSC in the active S‐phase of the cell cycle and detected a significantly lower proliferation rate of aged MSC compared to young MSC at early passages in culture (Figure 2b,c), although 50% of aged MSC were still able to enter in S‐phase under these conditions. To better quantify cell cycle progression in single human MSC from young and aged donors, we generated a lentiviral bicistronic reporter vector encoding fluorescent ubiquitination‐based cell cycle indicator probes (Fucci system). The lentiviral vector expresses mVenus‐hGeminin(1/110) fused to mCherry‐hCdt1(30/120) by the T2A peptide using an EF1α promoter that generates optimal levels of gene expression in primary cells (Pineda et al., 2016). These fluorescent reporters allow us to discriminate between three cell cycle phases as G1 by red fluorescence, G1/S by yellow fluorescence, and S/G2/M by green fluorescence. After a one‐hit transduction protocol, we monitored cell cycle transit up to 6 days in live imaging from young and aged MSC. Lentiviral transduction did not alter cell morphology or induced overt cell death in young or aged MSC (data not shown). Comparative analysis of cell cycle phases length between young and aged MSC revealed a significantly higher median duration of G1 in aged MSC compared to younger counterparts (71.2 vs. 28 hr, respectively) and a longer G1/S transition (11.44 vs. 1.16 hr, respectively) (Figure 2d–f). We also detected a higher median duration of S/G2/M phases in aged MSC compared to young MSC (16.4 vs. 9.56 hr) (Figure 2f). Of note, we noticed that a sizable fraction of aged MSC did not complete cell cycle within 6 days of analyses (data not shown), further indicating cell cycle delay in aged MSC.

When we analyzed the expression levels of cyclin‐dependent kinase (CDK) inhibitors associated with senescence in early passages MSC, we observed a significant increase in *CDKN1A *mRNA levels in aged MSC compared to younger MSC (Figure 2g) and only a trend toward differential expression of *CDKN2A *between the two MSC groups (Figure 2h). These data indicate that aged MSC were not fully arrested in the cell cycle at early passages but rather displayed a slowdown in their proliferation capacity.

Consistent with the induction of a senescence‐like state in early passages aged MSC, we observed a significant increase in the percentage of cells with a weak but detectable accumulation of SA‐β‐Gal cytosolic signal (Figure 2i,j). Similarly, when we evaluated lipofuscin accumulation by the biotin***‐***linked Sudan Black B (SBB) analogue staining (Evangelou et al., 2017), we found that aged MSC at early passages in culture display detectable lipofuscin granules compared to younger counterparts (Supporting information Figure S2a), further supporting the establishment of a “early‐senescence” phenotype in BM‐derived MSC from aged subjects.

### 2.3 Human BM‐derived aged MSC display modest accumulation of DNA damage, ROS, and activation of DDR

**Figure 3:**
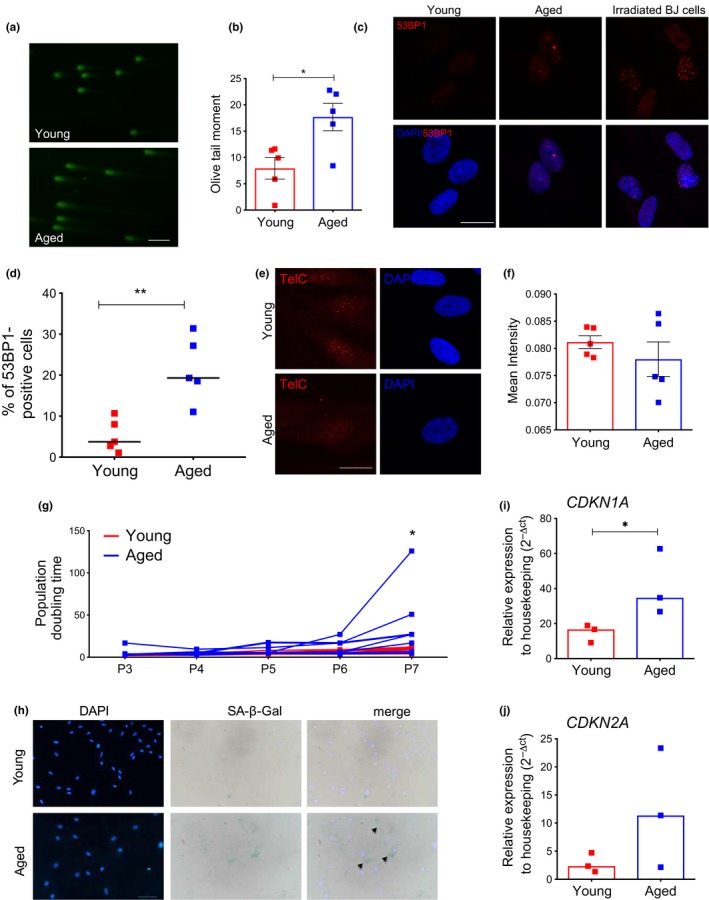
Aged MSC accumulate DNA damage and display modest DDR activation. (a) Representative fluorescence pictures of DNA damage in young and aged MSC as detected by comet assay; scale bar = 50 µm. (b) Quantification of alkaline comet assay carried out in young and aged MSC at early passages in culture (young, *n* = 5; aged, *n* = 5); up to 75 nuclei per sample were analyzed; histograms represent mean olive tail moment value ±*SEM* of young and aged MSC; *p*‐value was determined by Mann–Whitney test; *p  < 0.05. (c) Representative confocal pictures and (d) immunofluorescence quantification for 53BP1 foci‐positive cells in young and aged MSC at early passages in culture. Human BJ fibroblasts analyzed 2 hr post irradiation (20 Gy) were shown as positive control for 53BP1 nuclear staining in (c). Nuclei were counterstained with DAPI; scale bar = 20 µm (young, *n* = 5; aged, *n* = 5); *p*‐value was determined by Mann–Whitney test; **p  < 0.01. (e) Representative z‐stack confocal pictures of telomeric signal in young and aged MSC. Each red dot represents a telomere identified by a PNA probe against telomeric sequences. Scale bar = 20 µm. (f) Mean intensity of telomeric signal quantification was calculated with cell profiler (young, *n* = 5; aged, *n* = 5). Histograms represent mean values ±*SEM* of MSC samples analyzed (red = young; blue = aged). At least 20 nuclei were analyzed per sample with identical laser parameters. DAPI was used to stain nuclei. Scale bar = 20 µm. (g) Population doubling (PD) time of young (red lines) and aged (blue lines) MSC from passage 3 (P3) to passage 7 (P7); each line represents values of individual donors at each time point (young, *n* = 7; aged *n* = 7). *p*‐value was determined by Mann–Whitney test at passage 7; *p < 0.05. (h) Representative pictures of SA‐β‐Gal staining of MSC isolated from young and aged MSC at late passages (P10). Arrows indicate positive cells; scale bar = 200µm. (i–j) Relative mRNA expression of *CDKN1A *(i) and *CDKN2A *(j) measured by quantitative real‐time PCR at late passages in culture. Gene expression data are represented as 2^−△CT^ relative to *GUSB *housekeeping. Histograms indicate median 2^−△CT^ values, and each squared dot represents an individual MSC donor (red = young; blue = aged) (young, *n* = 3; aged, *n* = 3). *p*‐value was determined by Mann–Whitney test; *p  < 0.05

To further delve into the molecular mechanisms of the observed early‐senescence state of aged MSC, we evaluated the accumulation of physical DNA damage and the activation of the DDR pathway. We first performed alkaline comet assay to detect accumulation of both single‐ and double‐strand breaks in young and aged MSC. Quantitative analysis of a panel of MSC from young and aged donors indicated a significant increase in DNA damage accumulation in aged MSC (Figure 3a,b).

We next evaluated whether aged MSC had increased amount of oxidative stress. We measured the levels of reactive oxygen species (ROS) and reported a significant increase in ROS levels in aged MSC compared to younger MSC (Supporting information Figure S2b). The accumulation of cellular ROS was in line with the decreased expression levels of *FOXO4 *in aged MSC (Supporting information Figure S2c), a factor that has been previously shown to protect from oxidative stress during cellular homeostasis (Klotz et al., 2015). We also tested whether aged MSC display signs of oxidative DNA damage by measuring oxidation on guanosine residues by immunostaining with an antibody against 8‐oxo‐dG. Apparently, no increased oxidative DNA damage was detected in aged samples compared to young MSC (Supporting information Figure S2d). We next evaluated accumulation of DDR markers by immunofluorescence analysis and found a significant increase in the percentage of 53BP1 foci‐positive cells in MSC derived from aged donors (Figure 3c,d). Similarly, we observed a consistent activation of the phosphorylated form of the upstream DDR kinase ATM (pATMS1981) or the double‐strand break (DSB) marker *γ*H2A.X in MSC from aged donors (Supporting information Figure S2e,f). Moreover, as telomere shortening is a hallmark of aging and senescence, we characterized telomere length in our cohort of MSC by performing fluorescence in situ hybridization (FISH) with a PNA probe against telomeric sequences. Our quantitative imaging analysis revealed a trend toward less intense and possibly shorter telomeres, when comparing telomeric signals of aged MSC to young samples (Figure 3e,f). Altogether these data indicate that early passages aged MSC accumulate DNA damage and cellular ROS and display the activation of the DDR pathway.

We next assessed whether aged MSC reached full senescence in culture earlier than their younger MSC counterparts. We plated young and aged MSC in the same culture conditions and calculated the number of population doubling (PD) throughout several passages in culture. We observed that from passage 6–7, aged MSC showed a significant reduction in the proliferation ability compared to young MSC, indicative of an earlier induction of replicative senescence in the former group (Figure 3g). In agreement with this, late passages aged MSC displayed more intense accumulation of SA‐β‐Gal signal (Figure 3h) and higher levels of senescence‐associated cell cycle inhibitors *CDKN1A *and *CDKN2A *compared to younger counterparts (Figure 3i,j).

### 2.4 Aged MSC display a pro‐inflammatory SASP‐like program

Mesenchymal stromal cells exert potent anti‐inflammatory and immune‐suppressive functions on cells from the adaptive and innate immune system (Spaggiari & Moretta, 2013) and thus have therapeutic potential for various diseases in which inflammation plays a major role. As aging is associated with increased inflammation in the BM, dysfunctional immune‐suppressive MSC could contribute to the loss of homeostasis in the BM niche. We therefore investigated the immune‐regulatory capacity of aged MSC by measuring in vitro their effect on phytohemagglutinin (PHA)‐induced proliferation of allogeneic peripheral blood mononuclear cells (PBMC) from healthy donors (HD) (Supporting information Figure S3a). Whereas young MSC were able to strongly inhibit PHA‐induced PBMC proliferation at different MSC:PBMC ratios (1:2 to 1:20 and 1:200), aged MSC displayed a significantly reduced ability to inhibit proliferation of allogeneic PBMC when compared to younger counterparts (Supporting information Figure S3b). According to previous reports, MSC may exert their immunoregulatory activity through the production of chemokines including C‐X‐C motif chemokine 10 (CXCL10) (Bernardo & Fibbe, 2013; Hoogduijn et al., 2010) and the release of soluble mediators such as prostaglandin E2 (PGE2), transforming growth factor beta (TGF‐β), or indoleamine 2,3‐dioxygenase (IDO1). We therefore tested the basal expression levels of *CXCL10, *prostaglandin‐endoperoxide synthase 2 *(PTGS2) *as readout of PGE2 synthesis, *TGF‐β1 *and *IDO1 *in young and aged samples to identify molecular determinants of the reduced immunomodulatory function of aged MSC. Quantitative real‐time qPCR revealed that aged MSC displayed a dramatic decrease in *CXCL10 *mRNA levels compared to MSC derived from young donors at early passages (Supporting information Figure S3c), while no significant differences in the expression of *IDO1*, *PTGS2, *and *TGF‐β1 *between young and aged MSC were detected (Supporting information Figure S3d–f).

**Figure 4:**
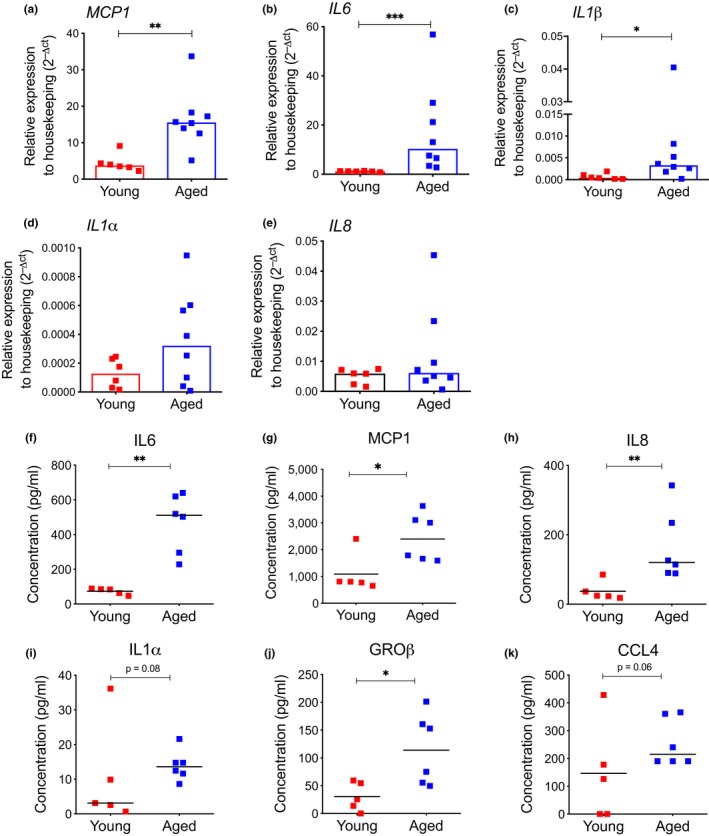
Aged MSC display activation of SASP. (a–e) Gene expression analysis for *MCP1, IL6, IL1β*, *IL1α, *and *IL8 *by quantitative real‐time PCR in young and aged MSC. Gene expression data are represented as 2^−△CT^ relative to *GUSB *housekeeping. Histograms indicate median 2^−△CT^ values (young, *n* = 6; aged, *n* = 8); p‐value was determined by Mann–Whitney test; ***p  < 0.001; **p  < 0.01; *p < 0.05. (f–k) Levels of IL6, MCP1, IL8, IL1α, GRO‐β, and CCL4 measured by Luminex assay in conditioned medium (CM) collected from young and aged MSC at early passages in culture. Values are represented as scatter dot plot; lines indicate median values (young, *n* = 5; aged, *n* = 6). *p*‐value was determined by Mann–Whitney test; **p  < 0.01; *p < 0.05

We then evaluated the levels of key inflammatory molecules in the plasma obtained from BM of young and aged donors. By exploiting the luminex technology, we reported a dramatic increase in IL6, CXCL8 (hereafter defined IL8), and MCP1 in the BM of elderly donors compared to young counterparts (Supporting information Figure S4a–c), whereas the levels of other cytokines including IL1α, TNF‐α, and IL1β were not differentially secreted in aged BM compared to young BM (Supporting information Figure S4d–f). Since the analyzed pro‐inflammatory cytokines are key components of the SASP (Acosta et al., 2008; Coppe et al., 2008), we assessed whether aged MSC could contribute to BM inflammation via SASP during aging. Quantitative real‐time PCR revealed a significant increase in *MCP1, IL6 *and *IL1β* at gene expression level in aged MSC compared to young MSC (Figure 4a–c). We also reported a trend toward increased mRNA levels of *IL1α *and *IL8 *in aged MSC (Figure 4d,e). To evaluate whether SASP factors are actively secreted by aged MSC, we collected conditioned medium (CM) from young and aged MSC at early passages in culture and analyzed the protein production of some key pro‐inflammatory cytokines. We found a robust increase in the secretion of several SASP molecules such as IL6, MCP1, IL8, IL1*α,* and Groβ and CCL4 in aged MSC compared to young MSC (Figure 4f–k). The induction of a SASP program was further exacerbated when analyzing late passages aged MSC compared to late passages younger counterparts (Supporting information Figure S4g–i).

Overall, these findings indicate that aged MSC display reduced immunomodulatory properties and increased levels of pro‐inflammatory molecules and suggest that aged stromal cells may contribute to alterations of the BM niche in a non cell‐autonomous fashion.

### 2.5 Aged MSC secretomes activate inflammatory genes in young HSPC

**Figure 5:**
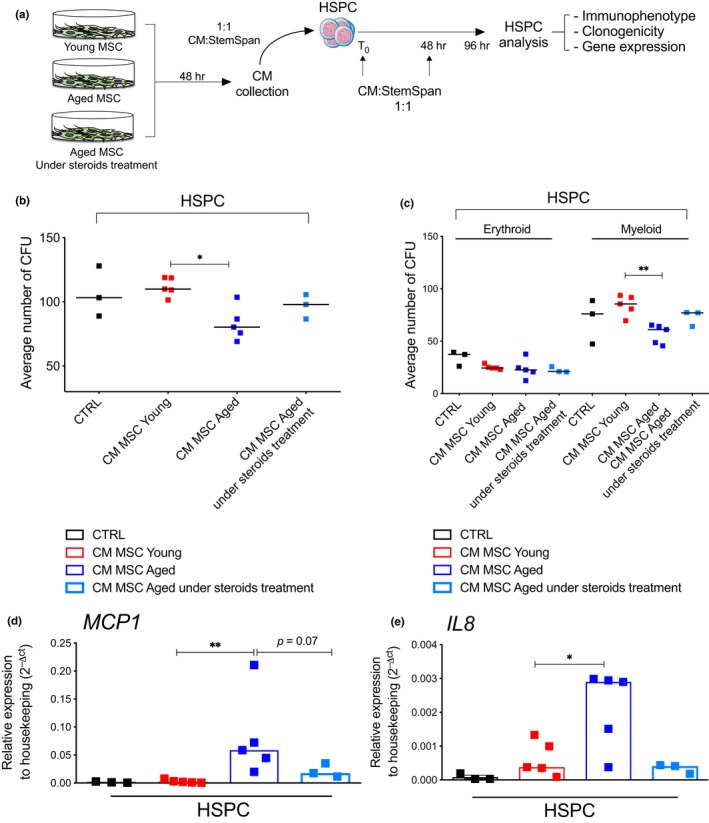
Paracrine effects of young and aged MSC secretomes on young HSPC. (a) Schematic representation of conditioned medium (CM) experiments with CM from young MSC, aged MSC and aged MSC under steroids treatment on cord blood (CB)‐derived CD34**^+^**cells (HSPC). Upon 96 hr of CM exposure, HSPC were analyzed for immunophenotype, clonogenicity, and gene expression. (b) Average number of HSPC colonies in methylcellulose analyzed at 96 hr post‐CM exposure. CM was collected from MSC derived from healthy young and aged donors, as well as from aged donors under chronic steroids treatment. Each dot represents average number of colonies generated from individual donors. CD34**^+^** cells (CTRL) grown without CM from MSC were used as control. (CTRL, *n* = 3; young, *n* = 5; aged, *n* = 5; aged under steroids treatment, *n* = 3). *p*‐value was determined by Mann–Whitney test; *p  < 0.05. (c) Distribution of erythroid and myeloid HSPC colonies in methylcellulose analyzed at 96 hr post exposure to CM from young MSC, aged MSC, and aged MSC from subjects under chronic steroids treatment. Each dot represents average number of colonies generated from individual donors. Lines indicate median values for each group. CD34**^+^** cells (CTRL) grown without CM from MSC were used as control. (CTRL, *n* = 3; young, *n* = 5; aged, *n* = 5; aged under steroids treatment, *n* = 3). *p *value was determined by Mann–Whitney test; **p < 0.01. (d–e) Gene expression analysis of *MCP1 *and *IL8 *by quantitative real‐time PCR in CB‐derived HSPC cultured for 96 hr either in the absence (CTRL) or in the presence of CM derived from young MSC, aged MSC, and aged MSC from subjects under chronic steroids treatment. Gene expression data are represented as 2^−△CT^ relative to *GUSB *housekeeping, histograms indicate median 2^−△CT^ values (CD34**^+^** CTRL *n* = 3; young, *n* = 5; aged, *n* = 5; aged under steroids treatment, *n* = 3); *p*‐value was determined by Mann–Whitney test; **p  < 0.01; *p < 0.05

We next investigated possible changes in the crosstalk between MSC and HSPC during aging. We first evaluated the expression levels of key factors involved in mediating supportive role of MSC in maintaining HSPC homeostasis in BM microenvironment, including *CXCL12*, *VCAM, VEGF, *and *ANGPT1*. None of these factors was significantly altered or diminished in aged MSC compared to those derived from young subjects (Supporting information Figure S4j–m). Given the above‐mentioned activation of a pro‐inflammatory transcriptional program in aged MSC (see Figure 4), we tested whether MSC secretomes could affect HSPC function. We collected conditioned medium (CM) from young and aged MSC and cultured umbilical cord blood (CB)‐derived CD34^+^ HSPC admixing hematopoietic stem cell media with MSC‐derived CM. We analyzed the effects of aged MSC secretome on the functionality of CD34^+^ cells by evaluating HSPC immune‐phenotype, clonogenic output in methylcellulose assays and gene expression changes (Figure 5a). As control we employed HSPC cultured in stem cell media in the absence of MSC‐derived CM. The mean percentages of CD34^+^ HSPC after 4 days of culture with CM derived from young or aged MSC were not significantly different from control HSPC (81.71% and 80.45%, respectively) (Supporting information Figure S5a). We also evaluated whether MSC‐secreted cytokines had an effect on the culture composition of HSPC, by using surface markers that identify primitive (CD90^+^CD133^+^), early (CD90^+^CD133^−^), or committed (CD90^−^CD133^−^) HSPC within the CD34‐+ as apex fraction in culture, as previously reported (Boitano et al., 2010; Doulatov, Notta, Laurenti, & Dick, 2012; Genovese et al., 2014). Immunophenotypic analysis of stem and progenitor cell markers did not identify any skewing in the culture composition over time when comparing HSPC cultured with MSC‐derived CM from young and aged donors compared to control (Supporting information Figure S5b). Whereas no differences were observed between the CFU ability of CD34^+^ HSPC control cells and CD34^+^ HSPC that received CM from young MSC, we observed a significant decrease in the total number of colonies generated from CD34^+^ HSPC cultured with CM derived from aged MSC, suggesting a paracrine effect of factors secreted by aged MSC on hematopoietic stem cell clonogenicity (Figure 5b). Of note, this detrimental effect on HSPC clonogenicity was more evident for myeloid rather than erythroid colonies (Figure 5c, Supporting information Figure S5c).

We next investigated whether inflammatory molecules secreted by aged MSC could activate an inflammatory transcriptional program in young HSPC. When we performed gene expression analysis of HSPC exposed to aged and young MSC‐enriched CM, we detected significant increased *MCP1 *and *IL8 *levels in young HSPC cells grown in the presence of CM derived from aged MSC CM compared either to CD34^+^ control cells or to the young counterparts (Figure 5d,e). Albeit not significant, we also reported a trend toward an increase for *IL1α *and *IL6 *mRNA levels in cells grown with CM derived from aged MSC (Supporting information Figure S5d,e).

We next tested the hypothesis that aged MSC from subjects with lower burden of chronic inflammation would have limited impact on HSPC functionality. We took advantage of MSC derived from 3 aged subjects under chronic treatment with steroids, known SASP inhibitors, and evaluated cytokine production and the non cell autonomous effects of their conditioned media on HSPC clonogenicity (Figure 5a). We found that MSC from subjects under steroids treatment displayed a reduced activation of key SASP components both at transcript (Supporting information Figure S5f–i) and protein levels (Supporting information Figure S5j–l) compared to aged MSC, without any difference in the induction of an ‘early‐senescence” phenotype as indicated by similar levels of *CDKN1A *(Supporting information Figure S5m) and MSC CFU‐F ability (data not shown) in comparison with MSC from aged healthy subjects. Interestingly, when we exposed HSPC to conditioned media derived from aged MSC of subjects under steroids treatment, the detrimental paracrine effect of SASP on HSPC clonogenicity was dampened dramatically when compared to CM derived from MSC of healthy untreated subjects of comparable age (Figure 5b,c; Supporting information Figure S5c). In agreement with this, HSPC exposed to CM collected from MSC derived from aged MSC under chronic steroids treatments displayed much lower levels of inflammatory cytokines when compared to HSPC exposed to CM of untreated aged subjects (Figure 5d,e; Supporting information Figure S5d,e).

Overall, these results indicate that aged MSC release SASP factors that propagate inflammatory signals to neighboring HSPC in a paracrine fashion and may in turn contribute to the reduced clonogenic capacity of recipient HSPC.

### 2.6 SASP inhibition in aged MSC rescues clonogenicity in young HSPC

**Figure 6:**
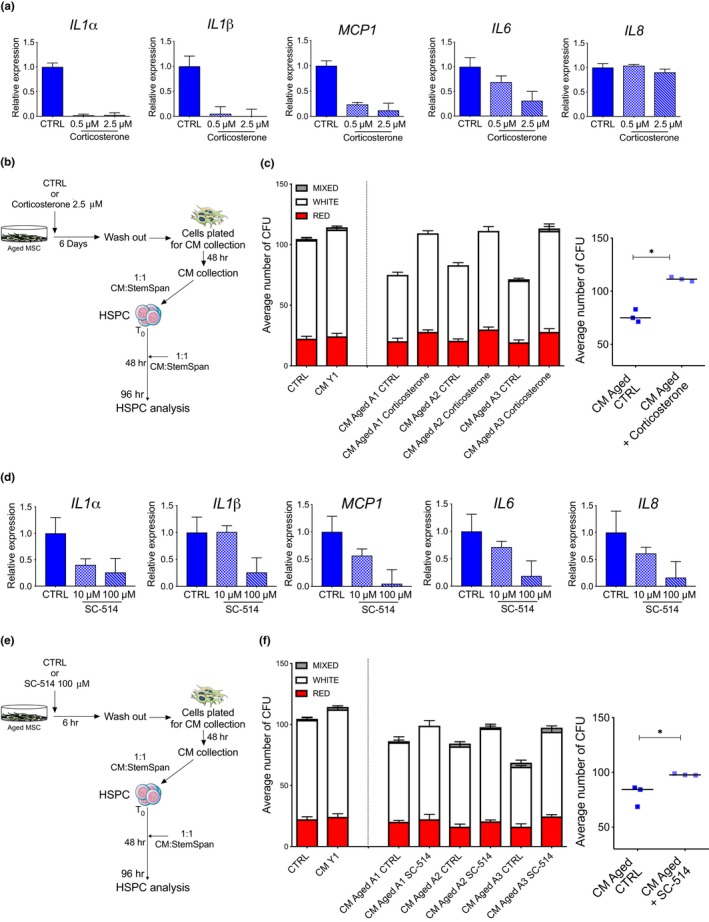
SASP inhibitors rescue the clonogenic impairment of young HSPC exposed to CM from aged MSC. (a) Relative mRNA levels of *IL1α, IL1β*, *MCP1, IL6, *and *IL8 *revealed by quantitative real‐time PCR in late passages aged MSC treated with vehicle CTRL (Ethanol), 0.5 µM, or 2.5 µM corticosterone for 6 days. *GUSB *was used as housekeeping gene; histograms represent fold change + *SD* relative to CTRL. (b) Experimental design to test the paracrine effect of corticosterone‐treated early passages aged MSC on young HSPC functionality. (c) Left panel. Number of HSPC colonies in methylcellulose analyzed at 96 hr postexposure to CM derived from aged MSC treated or not with 2.5 µM corticosterone for 6 days. Red, white, and light gray bars represent erythroid, myeloid, and mix colonies, respectively. CD34**^+^**cells grown without CM (CTRL) or with CM derived from young MSC were used as controls. Error bars indicate *SD* of three technical replicates for each individual sample. Right panel. Each dot represents average number of colonies generated from donors (aged CTRL, *n* = 3; aged treated with 2.5 µM corticosterone, *n* = 3). *p*‐value was determined by Mann–Whitney test; *p  < 0.05. (d) Gene expression analysis for *IL1α, IL1β*, *MCP1, IL6, *and *IL8 *measured by quantitative real‐time PCR in aged MSC treated for 6 hr with vehicle CTRL (DMSO), 10 µM, or 100 µM of the IKK‐2 inhibitor SC‐514. *GUSB *was used as housekeeping gene; histograms represent fold change + *SD* relative to CTRL. (e) Experimental design to test the paracrine effect of SC‐514‐treated early passages aged MSC on young HSPC functionality. (f) Left Panel. Number of HSPC colonies in methylcellulose analyzed at 96 hr postexposure to CM derived from aged MSC treated or not with 100 µM SC‐514 for 6 hr. Red, white, and light gray bars represent erythroid, myeloid, and mix colonies, respectively. CD34**^+^**cells grown without CM (CTRL) or with CM derived from young healthy MSC were used as controls. Error bars indicate *SD* of three technical replicates for each sample. Right Panel. Each dot represents average number of colonies generated from donors (aged CTRL, *n* = 3; aged treated with 100 µM SC‐514, *n* = 3). *p*‐value was determined by Mann–Whitney test; *p  < 0.05

In order to assess the direct effects of SASP inhibition on aged MSC and identify the molecular events that regulate the propagation of inflammation from MSC to HSPC, we employed ex vivo SASP inhibitor treatments on aged MSC. We first performed a 6 days long treatment with corticosterone, a glucocorticoid previously reported to dampen activation of SASP in senescent fibroblasts (Laberge et al., 2012). We measured the transcript levels of a panel of pro‐inflammatory SASP molecules and found a dose‐dependent suppression of SASP cytokines in aged MSC upon treatment (Figure 6a). We next collected conditioned media from 3 aged MSC left untreated or treated in the presence of corticosterone and cultured HSPC for 96 hr before assessing their clonogenic potential in methylcellulose assays (Figure 6b). Whereas HSPC exposed to CM from aged MSC displayed a reduced clonogenic potential compared to control or HSPC exposed to CM from young MSC, HSPC exposed to CM from corticosterone‐treated aged MSC gave rise to a significantly higher number of colonies (Figure 6c).

Because NFkB acts as a master regulator of the SASP transcriptional program in senescent cells (Chien et al., 2011), we tested the functional role of the NFkB pathway in aged MSC by employing a IKK‐2 Inhibitor (SC‐514), a suppressor of the NFkB‐dependent inflammatory transcriptional program (Kishore et al., 2003; Roman‐Blas & Jimenez, 2006). We detected a dose‐dependent transcriptional suppression of several key SASP molecules upon 6hr SC‐514 treatment in aged MSC (Figure 6d). We next tested the paracrine effects of CM derived from untreated and SC‐514‐treated aged MSC on young HSPC clonogenicity (Figure 6e). Similarly to corticosterone treatment, transient inhibition of the NFkB pathway in aged MSC significantly rescued the observed functional impairment of HSPC exposed to CM of control‐treated aged MSC (Figure 6f).

Overall, these findings indicate that SASP inhibition in aged MSC counteracts the detrimental effects of aged MSC secretomes on HSPC functionality.

## 3 DISCUSSION

Mesenchymal stromal cells are a functional component of the BM niche with a key role in supporting HSPC homeostasis (Kfoury & Scadden, 2015). Aging is characterized by alterations in niche components and factors that can contribute to HSPC dysfunction and to the onset and progression of many hematological diseases (Kumar, Godavarthy, & Krause, 2018). Here, we conducted extensive characterization of ex vivo expanded BM‐derived MSC from young and geriatric healthy donors and identified signs of early senescence that encompassed accumulation of SA‐β‐Gal and lipofuscin, increased level of physical DNA damage and activation of DDR, and induction of a pro‐inflammatory secretory phenotype in aged MSC at early culture passage. Importantly, aged MSC displayed reduced immunomodulatory capacity compared to younger counterparts and increased inflammation level. We also showed that the inflammatory program of aged MSC could be transferred in a paracrine fashion to HSPC, and likely contribute to age‐associated dysfunction of HSPC and bone marrow microenvironment inflammation. Finally, by employing SASP inhibitors treatment, we provide molecular evidence for the functional role of SASP in mediating HSPC dysfunction upon exposure to aged MSC secretomes.

Mesenchymal stromal cells are a rare and heterogeneous population present at very low frequency in both neonatal and adult tissues and represent only 0.01%–0.001% of nucleated cells in the BM. For this reason, MSC need to be extensively expanded in culture to be studied and/or used for clinical applications (Bernardo, Locatelli, & Fibbe, 2009; Kfoury & Scadden, 2015). We successfully derived MSC from several aged healthy donors thus indicating that advanced age does not preclude MSC generation and expansion. It is well accepted that MSC undergo replicative senescence after prolonged in vitro culture (Bernardo et al., 2007; Izadpanah et al., 2008; Wagner et al., 2008), reason for which we confined our molecular and cellular analysis within the first passages in culture. Unexpectedly, we report that, at early passages, aged MSC retain some capacity to actively progress to the S‐phase, although reduced compared to younger counterparts and display signs of early senescence rather than stable cell cycle arrest. Given that MSC require a few days to be established ex vivo, it is possible that, if present, the most senescent cells in aged BM may be counter‐selected during the first days of in vitro culture possibly due to the loss of their clonogenic capacity. Indeed, in our experiments aged MSC displayed a significantly reduced ability to form clones after plating as compared with young MSC. Another intriguing explanation for the absence of fully senescent MSC in the BM of elderly subjects is the possibility that senescent cells may still be cleared in vivo through the recruitment of immune cells via SASP factors (Lujambio, 2016).

The multipotent potential of MSC and their differentiation ability have been extensively dissected in mouse and human settings. In particular, several regulators of MSC biology including factors involved in bone formation have been shown to be dramatically reduced during aging, suggesting that aged MSC have a reduced differentiation capacity toward bone and are more primed to differentiate into the adipogenic lineage (Kim et al., 2017; Li et al., 2017; Ma et al., 2018). When testing the differentiation capacity of our aged human BM‐derived MSC, we did not detect any significant functional skewing of aged MSC toward adipocytes. Conversely, the induction of osteogenic transcription factors upon stimulation was reduced in aged MSC compared to younger counterparts, although MSC from young and aged donors formed similar amount of mineralized matrix in vitro. These data may suggest that the number of osteogenic progenitors within the MSC population rather than their quality is slightly affected with aging. Unfortunately, markers that have been proposed for the prospective isolation of osteogenic or adipogenic progenitors from the total MSC population are not exclusive and their role in defining osteo‐ or adipogenic progenitors remains controversial. However, it was reported that induction of cell senescence in stromal cells by genotoxic agents may impair bone differentiation in a p53‐dependent manner, thus supporting the concept that in physiologically aged MSC, the establishment of a presenescence state could contribute to slightly impair the induction of osteogenic genes upon differentiation cues (Despars, Carbonneau, Bardeau, Coutu, & Beausejour, 2013). Although donor variability may mask any differentiation skewing between young and aged MSC, data in the literature reporting on an unbalanced differentiation of aged MSCs may instead reflect a different MSC source (adipose tissues derived vs. BM‐derived) (Wegmeyer et al., 2013), different culture conditions (FBS vs. platelet lysate enriched culture medium), or the underlying pathologies of elderly subjects (Kawamura et al., 2018). Indeed, most data available to date on human aging of the stromal compartment are generated on MSC obtained during surgical procedures for treatment of different age‐related pathologies with strong immunological alterations and do not represent the biology of stromal bone marrow cells during physiological aging. Of note, in our cohort, in order to avoid any confounding effect due to underlying age‐related diseases, we excluded from the analysis patients with significant co‐morbidities and inflammatory conditions, including cancer and immunological defects.

Our findings indicate for the first time that early senescent BM‐derived MSC activate a robust pro‐inflammatory transcriptional program and display reduced immunomodulatory capacity toward cells of the adaptive immune system. As a consequence, aged MSC contribute to create and sustain an inflammatory milieu in the BM niche. These data indicate that, in concert with a cell‐autonomous dysfunction of aged MSC, extrinsic factors may as well alter MSC crosstalks with other BM cellular components. TGF‐β pathway has only recently emerged as a key mediator of the senescence associated with aged MSC derived from osteoarthritic bones (Kawamura et al., 2018).

When measuring the expression levels of *TGF‐β *in our aged MSC, we did not observe any significant increase compared to younger MSC. Instead, the levels of well‐established SASP factors, including IL1, IL6, IL8, and MCP1, were higher in aged MSC and actively secreted. In particular, MCP1 was among the most highly expressed SASP factors in aged MSC both at the transcript and protein levels. Consistent with these findings, MCP1 was reported to be epigenetically repressed in umbilical cord blood‐derived MSC and activated only when cells reach premature senescence due to prolonged culture conditions (Jin et al., 2016).

Here, we provide evidence that factors secreted by aged MSC drive an inflammatory transcriptional program in young HSPC and contribute to their clonogenic impairment. Over the past decade, a growing body of evidence revealed that inflammatory stimuli alter HSPC fate and functionality by affecting HSPC proliferation/quiescence status, differentiation potential, or HSPC‐niche interactions. In particular, it has been reported that chronic inflammation drives HSPC myeloid skewing and leads to HSPC exhaustion during aging (Essers et al., 2009; Haas et al., 2015; Pietras et al., 2016). Our data indicate that the secretome of aged MSC may as well contribute to boost inflammation in HSPC in a paracrine fashion. However, further investigations are needed to dissect the role of individual SASP factors secreted by aged MSC on HSPC biology and to determine whether chronic exposure of young HSPC to MSC‐derived inflammatory molecules may induce paracrine senescence in HSPC as previously described in other settings (Acosta et al., 2013). Based on their unique immunomodulatory and anti‐inflammatory properties, MSC have emerged as cellular therapy for a variety of clinical applications both in the context of hematopoietic stem cell transplantation (HSCT), including the treatment of acute graft‐versus‐host disease and their co‐infusion with HSPC to improve engraftment, and in the setting of regenerative medicine to blunt inflammatory responses and stimulate tissue repair (Bernardo & Locatelli, 2016). To date, more than 400 clinical trials are underway with MSC‐based therapies (Galipeau & Sensebe, 2018) and most of them enroll subjects of advanced age. Therefore, mechanistic understanding of transplanted aged MSC and their interplay with co‐infused HSPC is needed to assess safety and efficacy of MSC‐based therapies for elderly subjects in those settings where autologous MSC are employed. Alternatively third‐party, off‐the‐shelf young MSC may be considered for clinical application in geriatric patients when aged MSC functionality is proven to be impaired. Our data also indicate that the pretreatment of aged MSC with inhibitors of the SASP transcriptional program (e.g., steroids or NFkB inhibitor) rescues the detrimental effects of aged MSC secretomes on HSPC functionality. Along the same lines, a recent study showed that selective eradication of senescent stromal cells from the BM of aged mice was able to revert organ damage and functionally rejuvenate the hematopoietic compartment by reducing SASP factors and dampening chronic inflammation (Gao et al., 2018). Senolytic drugs have recently entered clinical testing in humans for chronic kidney disease (clinical trial NCT02848131), and changes in the proportion of senescent adipose‐tissue derived MSC will be considered a primary outcome for treatment efficacy (Kirkland & Tchkonia, 2017). If proven efficacious and with limited side effects, therapeutic strategies based on senescence modulation and/or SASP inhibition could be transformative not only in the setting of autologous and allogeneic HSCT, where a rejuvenated stromal compartment may positively influence engraftment kinetics and transplantation outcome, but also in the context of regenerative medicine. In this latter case, experimental approaches based on MSC rejuvenation may pave the way for novel bone and regenerative medicine MSC‐based applications that could have a major impact on the healthcare of the aging population.

## 4 EXPERIMENTAL PROCEDURES

### 4.1 Human samples and cell lines

Mesenchymal stromal cells were isolated from pediatric subjects (<18 years) (*n* = 4; range 5–15; median 7 years); young adults (18–35 years) (*n* = 6; range 25–32; median 27 years); and aged subjects (≥70) (*n* = 12; range 72–89; median 76 years). Pediatric and young subjects donated BM for hematopoietic stem cell transplantation (HSCT) at the San Raffaele Hospital, Milan. Aged MSC were derived from BM samples collected from *n* = 12 aged subjects who underwent hip replacement surgery at San Raffaele Hospital, Milan, after obtaining written informed consent. For all the samples, the clonogenic capacity at day 7 was tested (Figure 1b). As we did not observe any significant difference in the number of clones of MSC derived from pediatric and adult donors, we used these samples as a unifying “young” group and consistently analyzed up to 6 samples for subsequent experiments. In comparison with the “young” group, we randomly selected up to 8 aged samples for molecular and functional analyses on senescence features and consistently analyzed them throughout the manuscript for all the experiments. We excluded from our analyses all subjects with compromised immune functions and previous history of cancer or cancer treatments. We also excluded subjects that were positive to HBV, HCV, and HIV infections. The vast majority of the elderly subjects were diagnosed with hypertension. Frailty in geriatric assessment has only recently been recognized as a medical condition that identifies elderly individuals with unintentional weight loss, muscle weakness, a feeling of fatigue, slow walking speed, low levels of physical activity, chronic inflammation, depression, and social awkwardness. When we evaluated the subjects in our cohort by the Edmonton Frailty Scale (EFS; Perna et al., 2017), we only found one subject with an “apparently vulnerable” frailty score, who was not included in our molecular and functional studies. All the other subjects scored as “no frail,” most likely suggesting that our BM aspirates were collected from healthy aged subjects eligible for primary hip replacement surgery. Only for experiments shown in Figure 5 and Supporting information Figure S5, we employed MSC derived from 3 aged donors under chronic immunosuppressive therapy with steroids (*n* = 3; range 69–80; median 72 years). All experiments were performed in accordance with the San Raffaele Hospital‐approved research protocol TIGET09 and following ethical guidelines. A detailed list of pediatric, young adults, and aged MSC samples analyzed in each experimental panel is indicated in Supporting information Table S1. BJ normal human fibroblasts were purchased from ATCC and grown in DMEM supplemented with 10% FBS (EuroClone), 1% penicillin/streptomycin (Pen/Strep, EuroClone), and 2 mM l‐glutamine (l‐Glu, EuroClone). Senescence in BJ cells was induced by irradiation (20 Gy), and cells were analyzed for senescence markers two weeks post‐treatment. Irradiated BJ cells analyzed 2 hr post‐treatment were used a positive control for 53BP1 foci in Figure 3c.

### 4.2 Isolation and expansion of BM‐derived MSC

Mononuclear cells were isolated from BM aspirates by density gradient centrifugation using Lymphoprep technology (STEMCELL), and after CD34^+^ purification, CD34^−^ cells were plated in noncoated 75–150 cm^2^ tissue culture flasks (BD Falcon) at a density of 1 × 10^5^/cm^2^ in complete culture medium: DMEM low glucose (Gibco, Life Technologies) supplemented with 5% platelet lysate (PL), 1% penicillin/streptomycin (Pen/Strep, EuroClone), and 2 mM l‐glutamine (l‐Glu, EuroClone). After 48 hr in culture, nonadherent cells were removed and culture medium was replaced twice a week. MSC were harvested, after reaching ≥80% confluence, using Trypsin (EuroClone), and were propagated at a density of 4x10^3^ cells/cm^2^. MSC at passage 2–4 (P2–P4) were considered “early passages” MSC, while MSC at passage 10 (P10) were considered “late passages” MSC.

### 4.3 MSC CFU‐F ability and proliferative capacity

Fibroblast colony‐forming unit (CFU‐F) ability was assessed by examining the cultures at day 7 and 14 after initial seeding; clonogenic efficiency was calculated as number of colonies per 10^6^ MNC initially seeded for both young and aged subjects. Population doubling time (PDT) was determined at each passage for each MSC sample by using the formula: PDT= (duration × log(2))/log(Final concentration) ‐ log(Initial concentration) according to http://www.doubling-time.com/compute.php. Results were expressed as PDT from P3 to P7.

### 4.4 Flow cytometric analysis

Mesenchymal stromal cells were phenotypically characterized by flow cytometry at early passages to evaluate the presence of canonical MSC surface markers (CD90, CD105, and CD73), primitive MSC markers (CD146, CD271), and the absence of CD45, CD34, CD14, HLA‐DR, and CD31 markers. Cells were detached with trypsin and washed with PBS supplemented with 2% FBS. 1 × 10^5^ cells were incubated with the proper antibody mix for 10 min at RT in the dark. Unstained cells were used as negative control. All samples were run on BD FACSCanto II cytometer (BD Biosciences). At least events per sample were recorded. Analyses were performed using FlowJo software v9.3.2. Level of CD146 and CD271 expression was calculated as delta‐MFI relative to unstained control (MFI sample—MFI unstained control).

### 4.5 BrdU incorporation assay

For BrdU incorporation analysis, early passages MSC derived from young and aged donors were seeded at a density of 3 × 10^4^/well and grown on glass coverslips (Zeus super). Cells were incubated overnight with 30 μM BrdU (Sigma‐Aldrich) dissolved in sterile DMEM low glucose (Gibco, Life Technologies) supplemented with 5% PL, 1% Pen/Strep, and 2 mM l‐glu. Cell were then fixed with PFA 4% for 20 min RT, and incorporation of BrdU was evaluated by immunofluorescence with a primary antibody against BrdU (Sigma‐Aldrich) after cell permeabilization and DNA denaturation with 1‐hr DNAse treatment (RQ1, Promega). Detection of the primary antibody was performed using 488 Alexa Fluor® conjugated secondary antibody (Thermo fisher). Nuclei were counterstained with DAPI, and images were captured and analyzed using Widefields Zeiss Axio Observer.Z1 microscope. At least 60–100 nuclei per sample were used for quantification of BrdU‐positive cells. Representative confocal pictures were obtained using Leica TCS SP5 confocal laser microscope and visualized with Leica Application Suite (LAS) software (Leica Microsystems). Scale bar is indicated in figures and figure legends.

### 4.6 MSC differentiation capacity into osteoblasts and adipocytes

The osteogenic and adipogenic differentiation capacity of MSC was assessed at early passages by incubating cells with α‐MEM (Gibco, Life Technologies), 10% FBS (Mesenchymal Stem Cell Stimulation Supplement, StemCell Technologies), 1% Pen/Strep, and 2 mM l‐Glu supplemented with 10^−7^mol/L dexamethasone, 50 mg/ml l‐ascorbic acid; starting from day +7 of the culture 5 mmol/L β‐glycerol phosphate (all from Sigma‐Aldrich) was added to the medium. Adipogenic differentiation was evaluated at the same passages by incubating cells with α‐MEM, 10% FBS, 1% Pen/Strep, and 2 mM l‐Glu supplemented with 10^‐7^mol/L dexamethasone, 50 mg/ml l‐ascorbic acid, 100 mg/ml insulin, 50 mmol/L isobutyl methylxanthine, 0.5 mmol/L indomethacin, and 5 mmol/L β‐glycerol phosphate (all from Sigma‐Aldrich). Both osteogenic and adipogenic cultures were incubated for at least 3 weeks before evaluating differentiation. To detect osteogenic differentiation, cells were stained for calcium depositions with Alizarin Red S (Sigma‐Aldrich).

Adipogenic differentiation was evaluated through the morphological appearance of fat droplets stained with Oil Red O (Bio‐Optica). Differentiation capacity was evaluated by RT‐qPCR for the expression of adipogenic (*PPARγ*, *FABP4*, *LPL*) and osteogenic genes (*RUNX2*, *SPARC, COL1A2*).

### 4.7 SA‐β‐galactosidase assay

Induction of senescence of early passage MSC was assessed by staining MSC with a senescence β‐galactosidase (SA‐β‐Gal) Staining Kit (Cell Signaling Technology) according to manufacturer's instructions. In detail, MSC were plated on coverslips and after 24 hr fixed with PFA 4% for 20 min at RT and then incubated overnight with the SA‐β‐Gal staining solution (pH = 6.0) to reveal SA‐β‐Gal activity. Nuclei were then stained with DAPI (Sigma‐Aldrich), and images were acquired with a Nikon Eclipse inverted microscope. Cells displaying blue signal in the cytosol were counted as positive. At least 50 nuclei per sample were used for quantification of SA‐β‐Gal‐positive cells in each donor‐derived MSC. Scale bar is indicated in figures and figure legends.

### 4.8 Gene expression analysis

Total RNA was extracted using miRNeasy Micro kit (Qiagen) according to the manufacturer's instructions. cDNA synthesis was carried out with iScript cDNA synthesis kit (Bio‐Rad) using 30ng of RNA as starting material. 18.75 ng of cDNA was pre‐amplified using 1.25 µl of 1 μM pooled primer mix and TaqMan Pre‐amp Master Mix (Applied Biosystem) and then used for qPCR after 1:20 dilution. qPCR was performed using Fast Sybr Green Master Mix (Applied Biosystem). For differentiation analysis, RNA was extracted using RNeasy Micro Kit (Qiagen) according to manufacturer's instructions. DNase treatment was performed on RNA samples using RNase‐Free DNase kit (Qiagen), and cDNA was synthesized from 1 µg total RNA using the high capacity reverse transcription kit (Applied Biosystems). SYBR Green‐based quantitative PCR was performed using QuantiFast SYBR Green PCR Kit (Qiagen) starting from 5 ng of cDNA. All qPCR were run with a Viia7 real‐time PCR system (Applied Biosystems) with 40 cycles of amplification. Raw data (Ct values) were analyzed according to the comparative Ct method. Gene expression data were calculated as relative to housekeeping genes as *GUSB *or *ACTNB*, and for each sample plotted as 2^−△CT ^or as 2^−△△CT^ (fold change) as specified in figure and figure legends. Primers used for qPCR are listed in Supporting information Table S2.

### 4.9 In vitro PBMC proliferation assay with phytohemagglutinin

Peripheral blood mononuclear cells were purified by conventional Ficoll separation from heparinized samples obtained from healthy donors. PBMC proliferation either in the presence or in the absence of MSC was evaluated after stimulation with phytohemagglutinin (PHA‐P 4 µg/ml; Sigma‐Aldrich) in flat‐bottom 96‐well tissue culture plates (BD Falcon) containing RPMI 1640 medium (Gibco, Life Technologies) supplemented with 10% FBS. Briefly, after MSC seeding, 1 × 10^5^ PBMC per well were added at final MSC:PBMC ratios 1:2, 1:20, and 1:200, with PHA. After 3 days, co‐cultures were pulsed with ^3^H‐thymidine (1 µCi/well, specific activity 6.7 Ci/mmole, Perkin Elmer) and cells were harvested 18 hr later. ^3^H‐thymidine incorporation was measured with a MicroBeta TriLux 1,450 instrument (Perkin Elmer). Results were expressed as mean percentage of PBMC proliferation; we referred to PBMC proliferation alone (in the absence of MSC) as 100% and this percentage was used to normalize PBMC proliferation in the presence of MSC.

### 4.10 Alkaline COMET assay

To detect DNA damage in young and aged MSC, we employed alkaline comet assay. Specifically, after trypsinization, 2 × 10^3^ MSC were mixed with molten Comet LMAgarose (Trevigen, MD) at a ratio of 1:10 (v/v) and immediately pipetted onto CometSlides (Trevigen, MD) and placed at +4°C. Once solidified, the slides were immersed in prechilled Lysis Solution (Trevigen, MD) for 1 hr at +4°C. Following lysis, slides were immersed in freshly prepared Alkaline Unwinding Solution pH > 13 (300 mM NaOH, 1 mM EDTA) for 1 hr at +4°C and then electrophoresed in alkaline electrophoresis solution pH > 13 (300 mM NaOH, 1 mM EDTA) at 300 mA for 40 min. Slides were washed twice in ddH2O and fixed in 70% ethanol for 5 min. Comets were stained with SYBR Green for 30 min at RT. All steps were conducted in the dark to prevent additional DNA damage. Comets were analyzed using a Nikon Eclipse E600 microscope and a Nikon‐DS‐RI2 camera. Up to 75 nuclei for each individual donor were analyzed with CASP software to determine “Olive Tail Moments” of individual nuclei. Scale bar is indicated in figures and figure legends.

### 4.11 Intracellular ROS and oxidative DNA damage measurements

Reactive oxygen species levels were analyzed using the CellRox Reagents (Thermo Fisher) according to the manufacturer's instructions. In details, MSC were plated at a density of 4 × 10^4^ cells/cm^2^ and kept in culture for two days before ROS measurement. Cells were then incubated with 500 nM Cell Rox Reagent for 60 min at +37°C protected from light. During the last 15 min of staining, 5 nM SYNTOX Red Dead Cell stain was added to cell culture. Cells were washed with PBS, detached, and analyzed by flow cytometry using 488‐nm excitation for the CellROX Reagent and 639‐nm excitation for the SYTOX Red stain. Unstained samples were used as negative controls.

For 8‐oxo‐dG staining, 1.5 × 10^5^ cells were collected, washed with PBS supplemented with 2% FBS, and fixed with 1% PFA. Then, DNA was denatured by adding HCL 2N and cells permeabilized with PermBuffer 1× (Biolegend). Staining with the primary antibody against 8‐oxo‐dG (Trevigen) was performed overnight at +4°C and revealed with 488 Alexa Fluor® goat anti‐mouse IgG (Thermo Fisher). Cells stained with secondary antibody only were used as negative controls. Samples were run on BD FACSCanto II cytometer (BD Biosciences) and analyzed with FlowJo software v9.3.2.

### 4.12 Immunofluorescence analysis

Immunofluorescence was carried out on young and aged MSC at early passages in culture. Briefly, 3 × 10^4^ MSC were seeded in 24‐well plates on 10 mm round sterile coverslips. After 24 hr of culture, cells were fixed with 4% PFA for 20 min at RT and then permeabilized with 0.3% Triton‐X 100 in PBS for 10 min. After washing, coverslips were blocked in PBG (0.2% cold water fish gelatine, 0.5% BSA in PBS) for 30 min. Cells were then stained with primary antibodies 1 hr at RT followed by secondary antibodies Alexa488‐ or Alexa568‐conjugated goat anti‐mouse or goat anti‐rabbit IgGs (Thermo fisher). After DAPI staining (Sigma‐Aldrich), slides were mounted with Aqua‐Poly/mount (Polysciences Inc). Images were obtained with Leica TCS SP5 confocal laser microscope and visualized with Leica Application Suite (LAS) software (Leica Microsystems). Anti‐53BP1 polyclonal antibody was purchased from Bethyl Antibody (1:600), anti‐pATMS1981 (pATM) monoclonal antibody was purchased from Rockland (1:100), and anti‐γH2A.X monoclonal antibody was purchased from Biolegend (1:200). Scale bar is indicated in figures and figure legends.

### 4.13 Cloning of the Fucci lentiviral vector

The plasmid encoding for Fucci2A (pMK‐RQ‐Fucci‐2A) was provided by GeneArt (Thermo Fisher Scientific). To generate the lentiviral Fucci2A expression vector, the pMK‐RQ‐Fucci‐2A was digested with the restriction enzymes MluI (5’‐terminal) and SalI (3’‐terminal) to isolate the Fucci2A sequence from the plasmid backbone and then ligated into the pCCLsin.PPT.hEF1α.GFP backbone, previously digested with the same combination of restriction enzymes. The cloning was verified by sequencing from the EF1α promoter (5’‐TCA AGC CTC AGA CAG TGG TTC‐3’) to the WPRE sequence (5’‐AGC AGC GTA TCC ACA TAG CG‐3’). In the Fucci2A lentiviral reporter vector, the housekeeping elongation factor 1α (EF1α) promoter drives the expression of both mVenus‐hGem (1/110) and mCherry‐hCdt1 (30/120), and the presence of the T2A (2A self‐cleaving peptide) allows the formation of two independent proteins. Primers used in this study were synthesized by Sigma‐Aldrich. The restriction enzymes were supplied by New England Biolabs.

### 4.14 Cell cycle analysis by the Fucci2A system

Mesenchymal stromal cells derived from one young and two aged donors were transduced at a multiplicity of infection (MOI) of 20 for 16 hr. Cells were then detached, counted, and seeded at a density of 2 × 10^4^ cells in a 24‐well plate. Transduced cells were monitored with IncuCyte (Essen Biosciences) for 6 days, and time‐lapse imaging was acquired taking phase contrast, red, and green fluorescence signal recording images each 45 min. Cell cycle kinetics of transduced MSC from young and aged donors was analyzed by tracking single cells throughout animation, and single measurements were made for each image acquired by using the ImageJ analysis tool Pixel Inspection. The average red and green fluorescent intensities were recorded from every image throughout the entire time‐lapse animation. Specifically, 10 cells per each donor were analyzed, and for the comparison between young and aged MSC, we represented only cell cycle phases completed within 6 days of measurements and pooled together cell cycle lengths from the two aged MSC. Cell cycle kinetics was determined by plotting mean red and green fluorescent intensity over time using Prism software v8 (GraphPad Software Inc.).

### 4.15 SenTraGor staining—antibody‐enhanced detection of senescent cells

Early passages young (*n* = 4) and aged MSC (*n* = 6) were stained with SenTraGor Reagent according to manufacturer's instructions. In detail, 3 × 10^4^ MSC were plated on glass coverslips (Zeus super) and after 24 hr fixed with PFA 4% for 20 min at RT. Cells were washed once with 50% EtOH and once with 70% EtOH for 5 min at RT and incubated with filtered SenTraGor reagent for 8 min at RT. After several washing with 50% of EtOH at RT, cells were permeabilized with 0.5% Triton‐X and blocked with PBG 1× for 1 hr at RT. MSC were incubated with primary antibiotin antibody for 1 hr at RT, washed 3 times with PBG 1×, and then incubated with fluorescent secondary antibody Alexa568‐conjugated goat anti‐rabbit IgG (Thermo fisher). After DAPI staining (Sigma‐Aldrich), slides were mounted with Aqua‐Poly/mount (Polysciences Inc). Images were obtained with Leica TCS SP5 confocal laser microscope and visualized with Leica Application Suite (LAS) software (Leica Microsystems). Antibiotin antibody was purchased from Abcam (1:600). Senescent by Irradiation BJ cells were used as positive control for SenTraGor staining, while cells stained without SenTraGor reagent or in the absence of antibiotin primary antibody were used as negative controls. Scale bar is indicated in figures and figure legends.

### 4.16 SASP inhibitor treatments of aged MSC

Late passages aged MSC were treated with 0.5 µM or 2.5 µM corticosterone (Sigma‐Aldrich) every other day for 6 days in culture and then collected for gene expression analysis of SASP factors. Ethanol was used as vehicle control. Cells from 3 aged MSC at early passages in culture were treated every other day with 2.5 µM corticosterone for 6 days or vehicle control and then seeded for CM collection. After 48 hr, CM was collected and used to grow young HSPC as described.

Late passages aged MSC were treated for 6 hr with 10 µM or 100 µM of the IKK‐2 inhibitor SC‐514 (InSolution™ IKK‐2 Inhibitor, Merck) and then collected for gene expression analysis of SASP factors. DMSO was used as vehicle control. Cells from 3 aged donors at early passages in culture were treated with SC‐514 100 µM or vehicle control for 6 hr and then seeded for CM collection. After 48 hr, CM was collected and used to grow young HSPC as described.

### 4.17 Fluorescence in situ hybridization

To analyze telomeres in early passages young and aged MSC, we conducted fluorescence in situ hybridization on young and aged MSC grown and fixed on glass coverslips (Zeus super). To visualize telomeres, we employed a peptide nucleic acid (PNA) FISH probe (PNA Bio Inc) that was complementary to the TTAGGG repeats labeled with the fluorescent dye Cy3. Nuclei were stained with DAPI. Confocal sections were obtained with a Leica TCS SP5 confocal laser microscope by acquisition of optical *z*‐sections at different levels along the optical axis. Telomere length was analyzed by quantification of telomeric signal fluorescence intensities with ad hoc cell profiler‐based pipeline. Comparative fluorescence analyses were carried out in parallel with identical acquisition parameters. Scale bar is indicated in figures and figure legends.

### 4.18 Luminex assay

R&D system Luminex Kit was used to analyze cytokine's secretion both in sera obtained from BM aspirates of young and aged donors and in conditioned media (CM) derived from young and aged MSC. Sera from 6 BM of young adults and 8 BM of aged subjects were collected from BM aspirates and analyzed prior MSC derivation. For CM collection, MSC from young and aged donors were plated the day before at 4 × 10^5^ cells/well in a 6‐well plate in StemSpan (Stem Cell Technologies) without addition of other factors for 48 hr. Media were then collected, filtered with a 0.22µm PVDF filter (Millipore), and stored at −80°C to use for Luminex or CM experiments.

Customized Luminex plates were obtained to screen for: IL1α, IL8, IL6, MCP1, IL1β, TNF‐α, GROβ, and CCL4. Assays were run as per manufacturers’ instructions with standards and samples in technical duplicates. Data were acquired on a calibrated Bio‐Plex MAGPIX multiple reader system (Bio‐Rad) and visualized with Bio‐Plex manager Software.

### 4.19 Purification of CD34^+^ cells from cord blood

Human CD34^+^ cells were isolated through positive magnetic bead selection according to manufacturer's instructions (Miltenyi Biotec) from umbilical cord blood (CB) collected upon informed consent from healthy volunteers according to the Institutional Ethical Committee approved protocol (TIGET09). Alternatively, human CB‐derived CD34^+^ cells were purchased from Lonza (Lonza).

### 4.20 Conditioned medium experiments on CB‐derived CD34^+^ cells

To assess paracrine effects of young and aged MSC on CD34^+^ cells, we performed CM experiments culturing CB‐derived CD34^+^ cells with CM collected from either young or aged (healthy aged, aged under steroids treatment, aged treated with SC‐514 or with corticosterone, and vehicle controls) MSC mixed 1:1 with StemSpan medium (Stemcell) supplemented with TPO (20 ng/ml), SCF (100 ng/ml), 1% Pen/Strep (EuroClone), and 2 mM l‐Glu (EuroClone). Specifically, after thawing, CD34^+^ cells were plated in a 96‐well plate at a concentration of 4 × 10^3^ cells/ml and grown for 96 hr with the mixed 1:1 StemSpan/CM medium. A condition of CD34^+^ cells grown in Stemspan with TPO (20 ng/ml), SCF (100 ng/ml), 1% Pen/Strep (EuroClone), and 2 mM l‐Glu (EuroClone) without CM (CTRL) was also used to control for MSC‐derived effects. After 48 hr, medium was refreshed adding a new mix of StemSpan/CM medium to CD34^+^ cells. At 96 hr, cells were collected for immune‐phenotypic analysis, methylcellulose assay, and gene expression analysis.

For the immunophenotypic analysis, 2 × 10^4^ cells were evaluated for the percentage of primitive (CD90^+^/CD133^+^), early (CD90^−^/CD133^+^), and committed cells (CD90^−^/CD133^−^) within CD34^+^ cells by FACS staining. Cells were collected, washed with PBS supplemented with 2% FBS, fixed with 1% PFA, and incubated with the proper antibody mix for 20 min at +4°C in the dark. Unstained cells and fluorescence minus one (FMO) control were used as negative control for antibody staining. All samples were run on BD FACSCanto II cytometer (BD Biosciences) and analyzed with FlowJo software v9.3.2.

### 4.21 HSPC colony‐forming assays

For the colony‐forming unit (CFU) assays, HSPC that were cultured with MSC‐derived CM were reseeded onto a 35‐mm culture dish (Corning) at a density of 8 × 10^2^ cells per dish in triplicate in methylcellulose medium (MethoCult, Stemcell). After 14 days, hematopoietic cell‐derived colonies were counted under a light microscope. The colony types were identified and defined as myeloid (white), erythroid (red), or mixed (gray).

### 4.22 Statistical analysis

All data are presented as median values or mean ± *SD* or ±*SEM*, as indicated. Mann–Whitney test was used for comparisons between two experimental groups. Data were analyzed upon consulting with biostatisticians at CUSSB (University Center for Statistics in Biomedical Sciences) within the San Raffaele Hospital, Milan. Graphs were generated by Prism software v8 (GraphPad Software Inc.). *p *values <0.05 were considered significant (*p < 0.05; **p < 0.01; ***p < 0.001; ****p < 0.0001).

## CONFLICT OF INTEREST

None Declared.

## AUTHOR CONTRIBUTIONS

DG designed experiments, performed research, interpreted data, and wrote the manuscript. SC, LdV, VR, AC, EL, and SR performed research and interpreted data. GF and MO provided human aged bone marrow samples. MEB provided human pediatric and young adult bone marrow samples. MEB and RDM coordinated the study, supervised research, interpreted data, and wrote the manuscript.

## References

[acel12933-bib-0001] Acosta, J. C. , Banito, A. , Wuestefeld, T. , Georgilis, A. , Janich, P. , Morton, J. P. , … Gil, J. (2013). A complex secretory program orchestrated by the inflammasome controls paracrine senescence. Nature Cell Biology, 15(8), 978–990. 10.1038/ncb2784 23770676 PMC3732483

[acel12933-bib-0002] Acosta, J. C. , O'Loghlen, A. , Banito, A. , Guijarro, M. V. , Augert, A. , Raguz, S. , … Gil, J. (2008). Chemokine signaling via the CXCR2 receptor reinforces senescence. Cell, 133(6), 1006–1018. 10.1016/j.cell.2008.03.038 18555777

[acel12933-bib-0003] Adams, G. B. , Martin, R. P. , Alley, I. R. , Chabner, K. T. , Cohen, K. S. , Calvi, L. M. , … Scadden, T. (2007). Therapeutic targeting of a stem cell niche. Nature Biotechnology, 25(2), 238–243. 10.1038/nbt1281 17237769

[acel12933-bib-0004] Avanzini, M. A. , Bernardo, M. E. , Cometa, A. M. , Perotti, C. , Zaffaroni, N. , Novara, F. , … Locatelli, F. (2009). Generation of mesenchymal stromal cells in the presence of platelet lysate: A phenotypic and functional comparison of umbilical cord blood‐ and bone marrow‐ derived progenitors. Haematologica, 94(12), 1649–1660. 10.3324/haematol.2009.006171 19773264 PMC2791945

[acel12933-bib-0005] Bernardo, M. E. , & Fibbe, W. E. (2013). Mesenchymal stromal cells: Sensors and switchers of inflammation. Cell Stem Cell, 13(4), 392–402. 10.1016/j.stem.2013.09.006 24094322

[acel12933-bib-0006] Bernardo, M. E. , & Locatelli, F. (2016). Mesenchymal stromal cells in hematopoietic stem cell transplantation. Methods in Molecular Biology, 1416, 3–20. 10.1007/978-1-4939-3584-0_1 27236663

[acel12933-bib-0007] Bernardo, M. E. , Locatelli, F. , & Fibbe, W. E. (2009). Mesenchymal stromal cells. Annals of the New York Academy of Sciences, 1176, 101–117. 10.1111/j.1749-6632.2009.04607.x 19796238

[acel12933-bib-0008] Bernardo, M. E. , Zaffaroni, N. , Novara, F. , Cometa, A. M. , Avanzini, M. A. , Moretta, A. , … Locatelli, F. (2007). Human bone marrow derived mesenchymal stem cells do not undergo transformation after long‐term in vitro culture and do not exhibit telomere maintenance mechanisms. Cancer Research, 67(19), 9142–9149. 10.1158/0008-5472.CAN-06-4690 17909019

[acel12933-bib-0009] Boitano, A. E. , Wang, J. , Romeo, R. , Bouchez, L. C. , Parker, A. E. , Sutton, S. E. , … Cooke, M. P. (2010). Aryl hydrocarbon receptor antagonists promote the expansion of human hematopoietic stem cells. Science, 329(5997), 1345–1348. 10.1126/science.1191536 20688981 PMC3033342

[acel12933-bib-0010] Campisi, J. (2005). Senescent cells, tumor suppression, and organismal aging: Good citizens, bad neighbors. Cell, 120(4), 513–522. 10.1016/j.cell.2005.02.003 15734683

[acel12933-bib-0011] Chien, Y. , Scuoppo, C. , Wang, X. , Fang, X. , Balgley, B. , Bolden, J. E. , … Lowe, S. W. (2011). Control of the senescence‐associated secretory phenotype by NF‐kappaB promotes senescence and enhances chemosensitivity. Genes & Development, 25(20), 2125–2136. 10.1101/gad.17276711 21979375 PMC3205583

[acel12933-bib-0012] Coppe, J. P. , Patil, C. K. , Rodier, F. , Sun, Y. , Munoz, D. P. , Goldstein, J. , … Campisi, J. (2008). Senescence‐associated secretory phenotypes reveal cell‐nonautonomous functions of oncogenic RAS and the p53 tumor suppressor. PLoS Biology, 6(12), 2853–2868. 10.1371/journal.pbio.0060301 19053174 PMC2592359

[acel12933-bib-0013] Despars, G. , Carbonneau, C. L. , Bardeau, P. , Coutu, D. L. , & Beausejour, C. M. (2013). Loss of the osteogenic differentiation potential during senescence is limited to bone progenitor cells and is dependent on p53. PLoS One, 8(8), e73206 10.1371/journal.pone.0073206 24009740 PMC3756945

[acel12933-bib-0014] Dominici, M. , Le Blanc, K. , Mueller, I. , Slaper‐Cortenbach, I. , Marini, F. , Krause, D. , … Horwitz,, (2006). Minimal criteria for defining multipotent mesenchymal stromal cells. The International Society for Cellular Therapy position statement. Cytotherapy, 8(4), 315–317. 10.1080/14653240600855905 16923606

[acel12933-bib-0015] Doulatov, S. , Notta, F. , Laurenti, E. , & Dick, J. E. (2012). Hematopoiesis: A human perspective. Cell Stem Cell, 10(2), 120–136. 10.1016/j.stem.2012.01.006 22305562

[acel12933-bib-0016] Essers, M. A. , Offner, S. , Blanco‐Bose, W. E. , Waibler, Z. , Kalinke, U. , Duchosal, M. A. , & Trumpp, A. (2009). IFNalpha activates dormant haematopoietic stem cells in vivo. Nature, 458(7240), 904–908. 10.1038/nature07815 19212321

[acel12933-bib-0017] Evangelou, K. , Lougiakis, N. , Rizou, S. V. , Kotsinas, A. , Kletsas, D. , Munoz‐Espin, D. , … Gorgoulis, V. G. (2017). Robust, universal biomarker assay to detect senescent cells in biological specimens. Aging Cell, 16(1), 192–197. 10.1111/acel.12545 28165661 PMC5242262

[acel12933-bib-0018] Farr, J. N. , Xu, M. , Weivoda, M. M. , Monroe, D. G. , Fraser, D. G. , Onken, J. L. , … Khosla, S. (2017). Targeting cellular senescence prevents age‐related bone loss in mice. Nature Medicine, 23(9), 1072–1079. 10.1038/nm.4385 PMC5657592 28825716

[acel12933-bib-0019] Galipeau, J. , & Sensebe, L. (2018). Mesenchymal stromal cells: Clinical challenges and therapeutic opportunities. Cell Stem Cell, 22(6), 824–833. 10.1016/j.stem.2018.05.004 29859173 PMC6434696

[acel12933-bib-0020] Gao, B. , Lin, X. , Jing, H. , Fan, J. , Ji, C. , Jie, Q. , … Yang, L. (2018). Local delivery of tetramethylpyrazine eliminates the senescent phenotype of bone marrow mesenchymal stromal cells and creates an anti‐inflammatory and angiogenic environment in aging mice. Aging Cell, 17(3), e12741 10.1111/acel.12741 29488314 PMC5946084

[acel12933-bib-0021] Geiger, H. , de Haan, G. , & Florian, M. C. (2013). The ageing haematopoietic stem cell compartment. Nature Reviews Immunology, 13(5), 376–389. 10.1038/nri3433 23584423

[acel12933-bib-0022] Genovese, P. , Schiroli, G. , Escobar, G. , Tomaso, T. D. , Firrito, C. , Calabria, A. , … Naldini, L. (2014). Targeted genome editing in human repopulating haematopoietic stem cells. Nature, 510(7504), 235–240. 10.1038/nature13420 24870228 PMC4082311

[acel12933-bib-0023] Haas, S. , Hansson, J. , Klimmeck, D. , Loeffler, D. , Velten, L. , Uckelmann, H. , … Essers, M. A. (2015). Inflammation‐induced emergency megakaryopoiesis driven by hematopoietic stem cell‐like megakaryocyte progenitors. Cell Stem Cell, 17(4), 422–434. 10.1016/j.stem.2015.07.007 26299573

[acel12933-bib-0024] Hoogduijn, M. J. , Popp, F. , Verbeek, R. , Masoodi, M. , Nicolaou, A. , Baan, C. , & Dahlke, M. H. (2010). The immunomodulatory properties of mesenchymal stem cells and their use for immunotherapy. International Immunopharmacology, 10(12), 1496–1500. 10.1016/j.intimp.2010.06.019 20619384

[acel12933-bib-0025] Ingo, D. M. , Redaelli, D. , Rossella, V. , Perini, O. , Santoleri, L. , Ciceri, F. , … Bernardo, M. E. (2016). Bone marrow‐derived CD34(‐) fraction: A rich source of mesenchymal stromal cells for clinical application. Cytotherapy, 18(12), 1560–1563. 10.1016/j.jcyt.2016.08.011 27742233

[acel12933-bib-0026] Izadpanah, R. , Kaushal, D. , Kriedt, C. , Tsien, F. , Patel, B. , Dufour, J. , & Bunnell, B. A. (2008). Long‐term in vitro expansion alters the biology of adult mesenchymal stem cells. Cancer Research, 68(11), 4229–4238. 10.1158/0008-5472.CAN-07-5272 18519682 PMC2713721

[acel12933-bib-0027] Jin, H. J. , Lee, H. J. , Heo, J. , Lim, J. , Kim, M. , Kim, M. K. , … Kim, S. W. (2016). Senescence‐Associated MCP‐1 Secretion Is Dependent on a Decline in BMI1 in Human Mesenchymal Stromal Cells. Antioxidants & Redox Signaling, 24(9), 471–485. 10.1089/ars.2015.6359 26573462 PMC4800271

[acel12933-bib-0028] Kawamura, H. , Nakatsuka, R. , Matsuoka, Y. , Sumide, K. , Fujioka, T. , Asano, H. , … Sonoda, Y. (2018). TGF‐beta signaling accelerates senescence of human bone‐derived CD271 and SSEA‐4 double‐positive mesenchymal stromal cells. Stem Cell Reports, 10(3), 920–932. 10.1016/j.stemcr.2018.01.030 29478902 PMC5918367

[acel12933-bib-0029] Kfoury, Y. , & Scadden, D. T. (2015). Mesenchymal cell contributions to the stem cell niche. Cell Stem Cell, 16(3), 239–253. 10.1016/j.stem.2015.02.019 25748931

[acel12933-bib-0030] Kim, H. N. , Chang, J. , Shao, L. , Han, L. , Iyer, S. , Manolagas, S. C. , … Almeida, M. (2017). DNA damage and senescence in osteoprogenitors expressing Osx1 may cause their decrease with age. Aging Cell, 16(4), 693–703. 10.1111/acel.12597 28401730 PMC5506444

[acel12933-bib-0031] Kirkland, J. L. , & Tchkonia, T. (2017). Cellular senescence: A translational perspective. EBioMedicine, 21, 21–28. 10.1016/j.ebiom.2017.04.013 28416161 PMC5514381

[acel12933-bib-0032] Kishore, N. , Sommers, C. , Mathialagan, S. , Guzova, J. , Yao, M. , Hauser, S. , … Tripp, C. S. (2003). A selective IKK‐2 inhibitor blocks NF‐kappa B‐dependent gene expression in interleukin‐1 beta‐stimulated synovial fibroblasts. Journal of Biological Chemistry, 278(35), 32861–32871. 10.1074/jbc.M211439200 12813046

[acel12933-bib-0033] Klotz, L. O. , Sanchez‐Ramos, C. , Prieto‐Arroyo, I. , Urbanek, P. , Steinbrenner, H. , & Monsalve, M. (2015). Redox regulation of FoxO transcription factors. Redox Biology, 6, 51–72. 10.1016/j.redox.2015.06.019 26184557 PMC4511623

[acel12933-bib-0034] Kumar, R. , Godavarthy, P. S. , & Krause, D. S. (2018). The bone marrow microenvironment in health and disease at a glance. Journal of Cell Science, 131(4), 10.1242/jcs.201707 29472498

[acel12933-bib-0035] Laberge, R. M. , Zhou, L. , Sarantos, M. R. , Rodier, F. , Freund, A. , de Keizer, P. L. , … Campisi, J. (2012). Glucocorticoids suppress selected components of the senescence‐associated secretory phenotype. Aging Cell, 11(4), 569–578. 10.1111/j.1474-9726.2012.00818.x 22404905 PMC3387333

[acel12933-bib-0036] Li, H. , Liu, P. , Xu, S. , Li, Y. , Dekker, J. D. , Li, B. , … Guo, X. (2017). FOXP1 controls mesenchymal stem cell commitment and senescence during skeletal aging. Journal of Clinical Investigation, 127(4), 1241–1253. 10.1172/JCI89511 28240601 PMC5373872

[acel12933-bib-0037] Liu, H. , Xia, X. , & Li, B. (2015). Mesenchymal stem cell aging: Mechanisms and influences on skeletal and non‐skeletal tissues. Experimental Biology and Medicine, 240(8), 1099–1106. 10.1177/1535370215591828 26088863 PMC4935286

[acel12933-bib-0038] Lujambio, A. (2016). To clear, or not to clear (senescent cells)? That is the question. BioEssays, 38(Suppl 1), S56–64. 10.1002/bies.201670910 27417123

[acel12933-bib-0039] Ma, Y. , Qi, M. , An, Y. , Zhang, L. , Yang, R. , Doro, D. H. , … Jin, Y. (2018). Autophagy controls mesenchymal stem cell properties and senescence during bone aging. Aging Cell, 17(1), e12709 10.1111/acel.12709 PMC5770781 29210174

[acel12933-bib-0040] Mabuchi, Y. , Morikawa, S. , Harada, S. , Niibe, K. , Suzuki, S. , Renault‐Mihara, F. , … Matsuzaki, Y. (2013). LNGFR(+)THY‐1(+)VCAM‐1(hi+) cells reveal functionally distinct subpopulations in mesenchymal stem cells. Stem Cell Reports, 1(2), 152–165. 10.1016/j.stemcr.2013.06.001 24052950 PMC3757748

[acel12933-bib-0041] Mendelson, A. , & Frenette, P. S. (2014). Hematopoietic stem cell niche maintenance during homeostasis and regeneration. Nature Medicine, 20(8), 833–846. 10.1038/nm.3647 PMC4459580 25100529

[acel12933-bib-0042] Mendez‐Ferrer, S. , Michurina, T. V. , Ferraro, F. , Mazloom, A. R. , Macarthur, B. D. , Lira, S. A. , … Frenette, P. S. (2010). Mesenchymal and haematopoietic stem cells form a unique bone marrow niche. Nature, 466(7308), 829–834. 10.1038/nature09262 20703299 PMC3146551

[acel12933-bib-0043] Morrison, S. J. , & Scadden, D. T. (2014). The bone marrow niche for haematopoietic stem cells. Nature, 505(7483), 327–334. 10.1038/nature12984 24429631 PMC4514480

[acel12933-bib-0044] Perna, S. , Francis, M. D. , Bologna, C. , Moncaglieri, F. , Riva, A. , Morazzoni, P. , … Rondanelli, M. (2017). Performance of Edmonton Frail Scale on frailty assessment: Its association with multi‐dimensional geriatric conditions assessed with specific screening tools. BMC Geriatrics, 17(1), 2 10.1186/s12877-016-0382-3 28049443 PMC5209899

[acel12933-bib-0045] Pietras, E. M. , Mirantes‐Barbeito, C. , Fong, S. , Loeffler, D. , Kovtonyuk, L. V. , Zhang, S. , … Passegue, E. (2016). Chronic interleukin‐1 exposure drives haematopoietic stem cells towards precocious myeloid differentiation at the expense of self‐renewal. Nature Cell Biology, 18(6), 607–618. 10.1038/ncb3346 27111842 PMC4884136

[acel12933-bib-0046] Pineda, G. , Lennon, K. M. , Delos Santos, N. P. , Lambert‐Fliszar, F. , Riso, G. L. , Lazzari, E. , … Jamieson, C. H. (2016). Tracking of normal and malignant progenitor cell cycle transit in a defined Niche. Scientific Reports, 6, 23885 10.1038/srep23885 27041210 PMC4819192

[acel12933-bib-0047] Roman‐Blas, J. A. , & Jimenez, S. A. (2006). NF‐kappaB as a potential therapeutic target in osteoarthritis and rheumatoid arthritis. Osteoarthritis Cartilage, 14(9), 839–848. 10.1016/j.joca.2006.04.008 16730463

[acel12933-bib-0048] Sacchetti, B. , Funari, A. , Michienzi, S. , Di Cesare, S. , Piersanti, S. , Saggio, I. , … Bianco, P. (2007). Self‐renewing osteoprogenitors in bone marrow sinusoids can organize a hematopoietic microenvironment. Cell, 131(2), 324–336. 10.1016/j.cell.2007.08.025 17956733

[acel12933-bib-0049] Spaggiari, G. M. , & Moretta, L. (2013). Cellular and molecular interactions of mesenchymal stem cells in innate immunity. Immunology and Cell Biology, 91(1), 27–31. 10.1038/icb.2012.62 23146943

[acel12933-bib-0050] Tormin, T. F. , Gimenes, D. T. , Silva, L. G. , Ruggiero, R. , Richter, E. M. , Ferreira, V. S. , & Munoz, R. A. (2010). Direct amperometric determination of tert‐butylhydroquinone in biodiesel. Talanta, 82(4), 1599–1603. 10.1016/j.talanta.2010.07.011 20801379

[acel12933-bib-0051] Wagner, W. , Horn, P. , Castoldi, M. , Diehlmann, A. , Bork, S. , Saffrich, R. , … Ho, A. D. (2008). Replicative senescence of mesenchymal stem cells: A continuous and organized process. PLoS One, 3(5), e2213 10.1371/journal.pone.0002213 18493317 PMC2374903

[acel12933-bib-0052] Wegmeyer, H. , Broske, A. M. , Leddin, M. , Kuentzer, K. , Nisslbeck, A. K. , Hupfeld, J. , … Neubauer, M. (2013). Mesenchymal stromal cell characteristics vary depending on their origin. Stem Cells and Development, 22(19), 2606–2618. 10.1089/scd.2013.0016 23676112 PMC3780294

