# Supplementary material for: An early‐senescence state in aged mesenchymal stromal cells contributes to hematopoietic stem and progenitor cell clonogenic impairment through the activation of a pro‐inflammatory program
Source: Aging Cell. 2019 Mar 3;18(3):e12933. doi: 10.1111/acel.12933 (PMC6516180; doi:10.1111/acel.12933)

Figure Supplementary 1 Gnani D. et al.

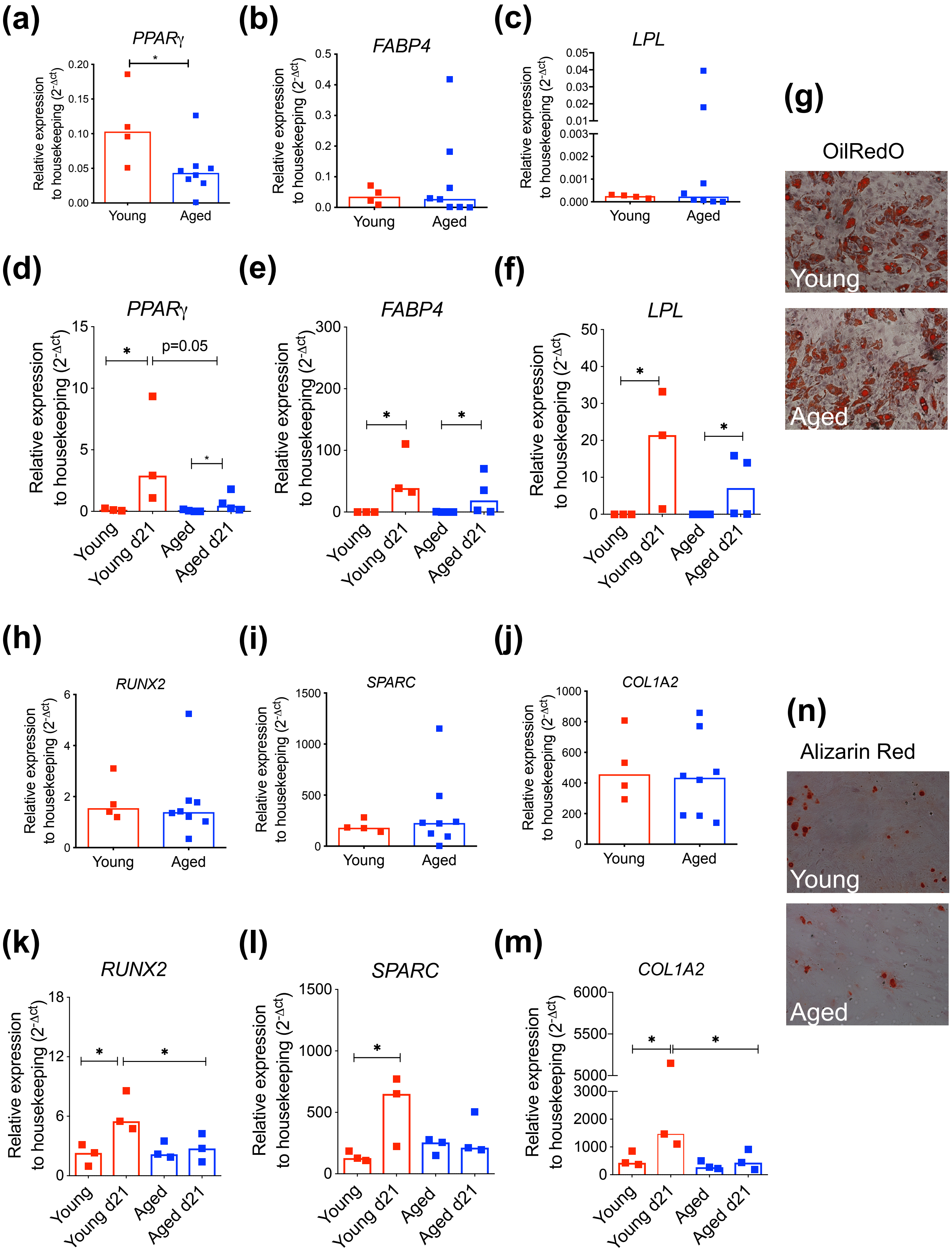

Figure Supplementary 2 Gnani D. et al.

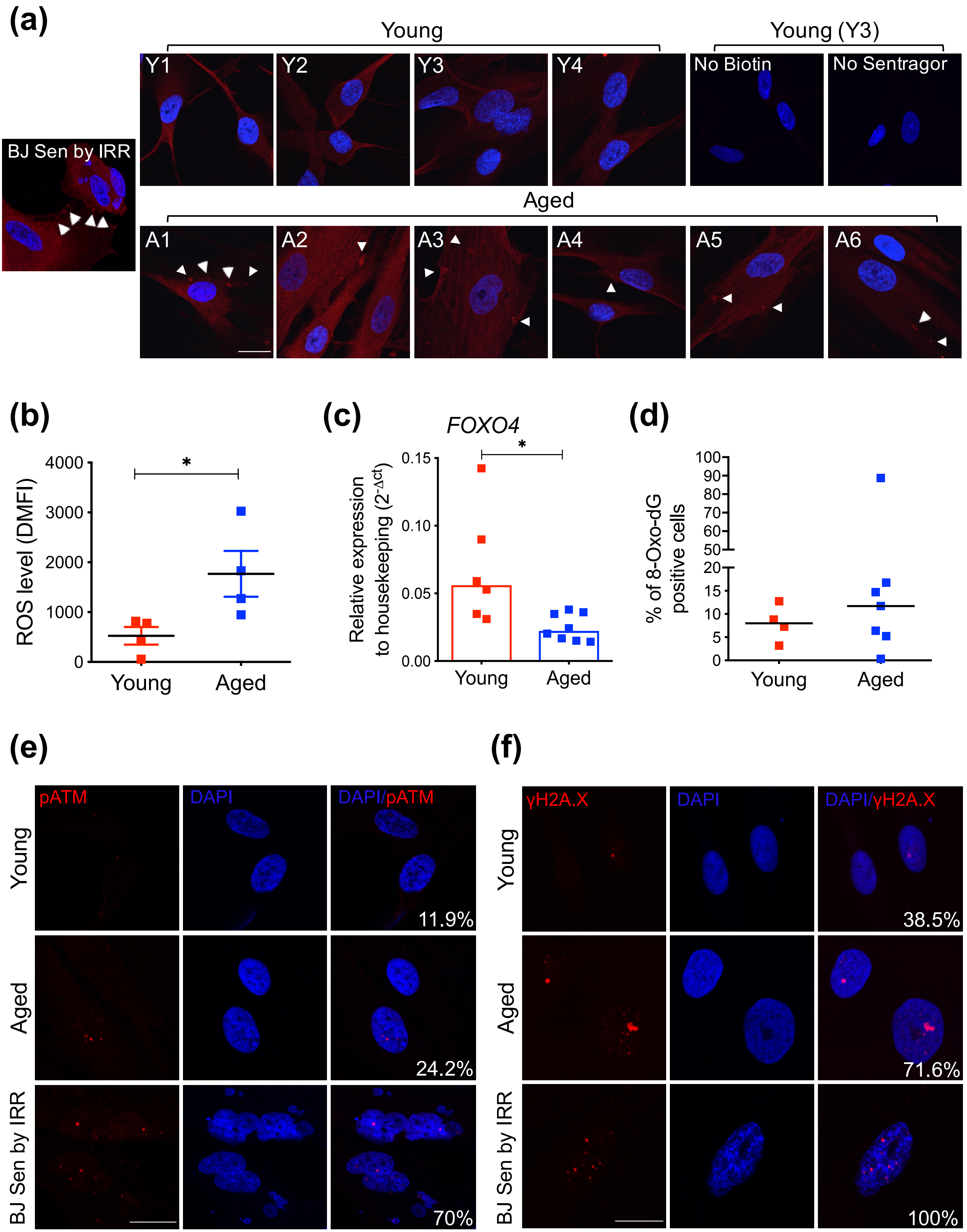

Figure Supplementary 3 Gnani D. et al.

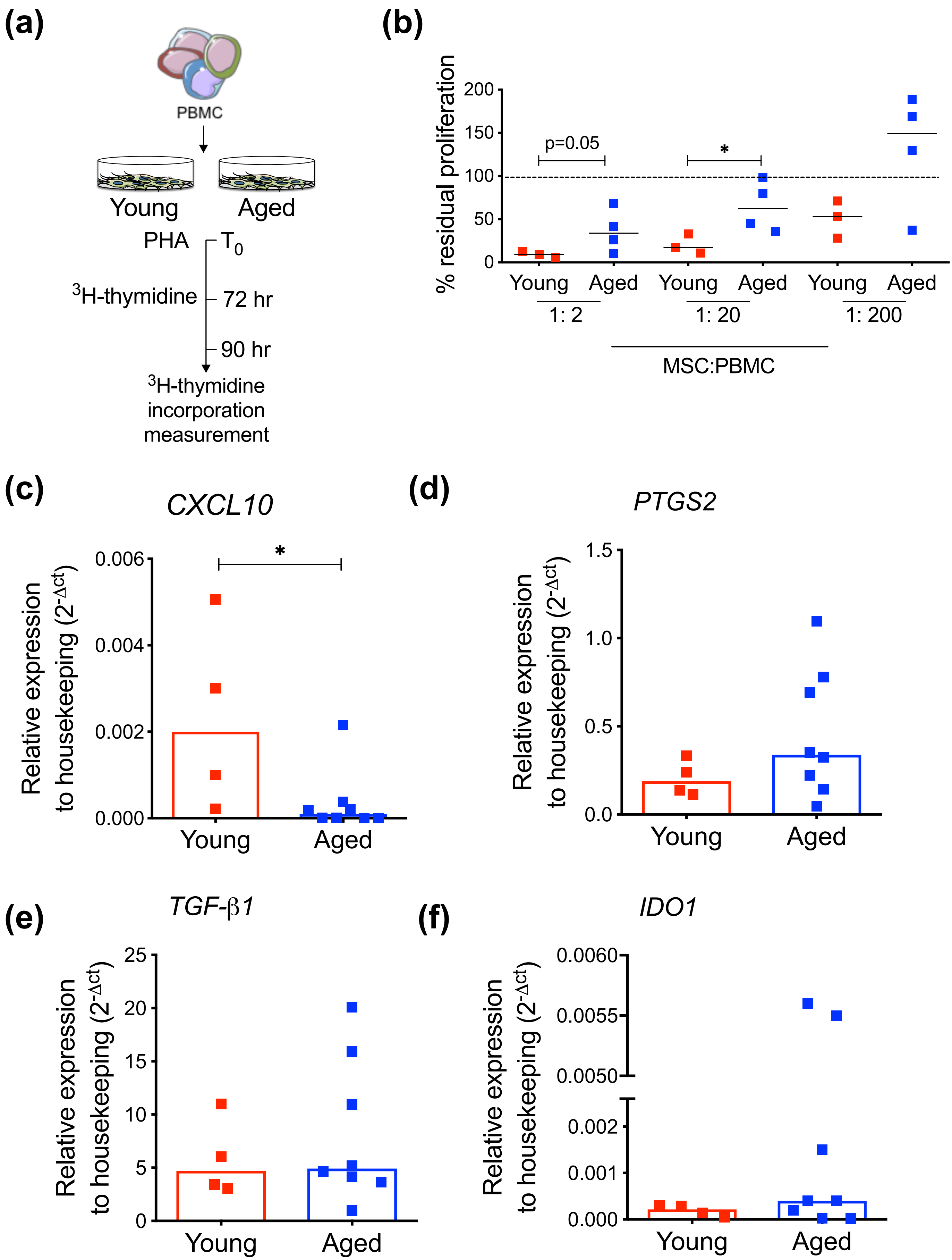

Figure Supplementary 4 Gnani D. et al.

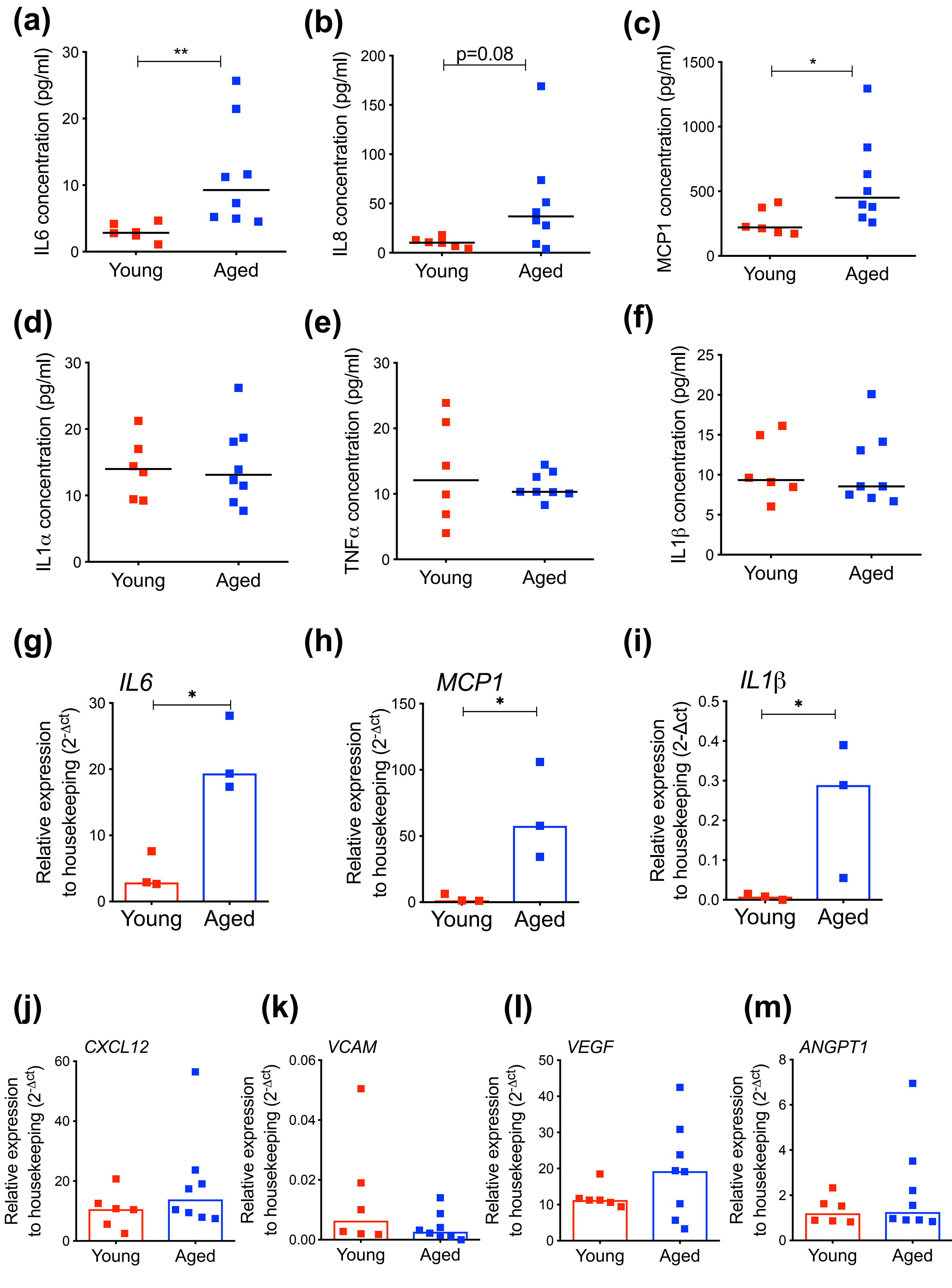

Figure Supplementary 5 Gnani D. et al.

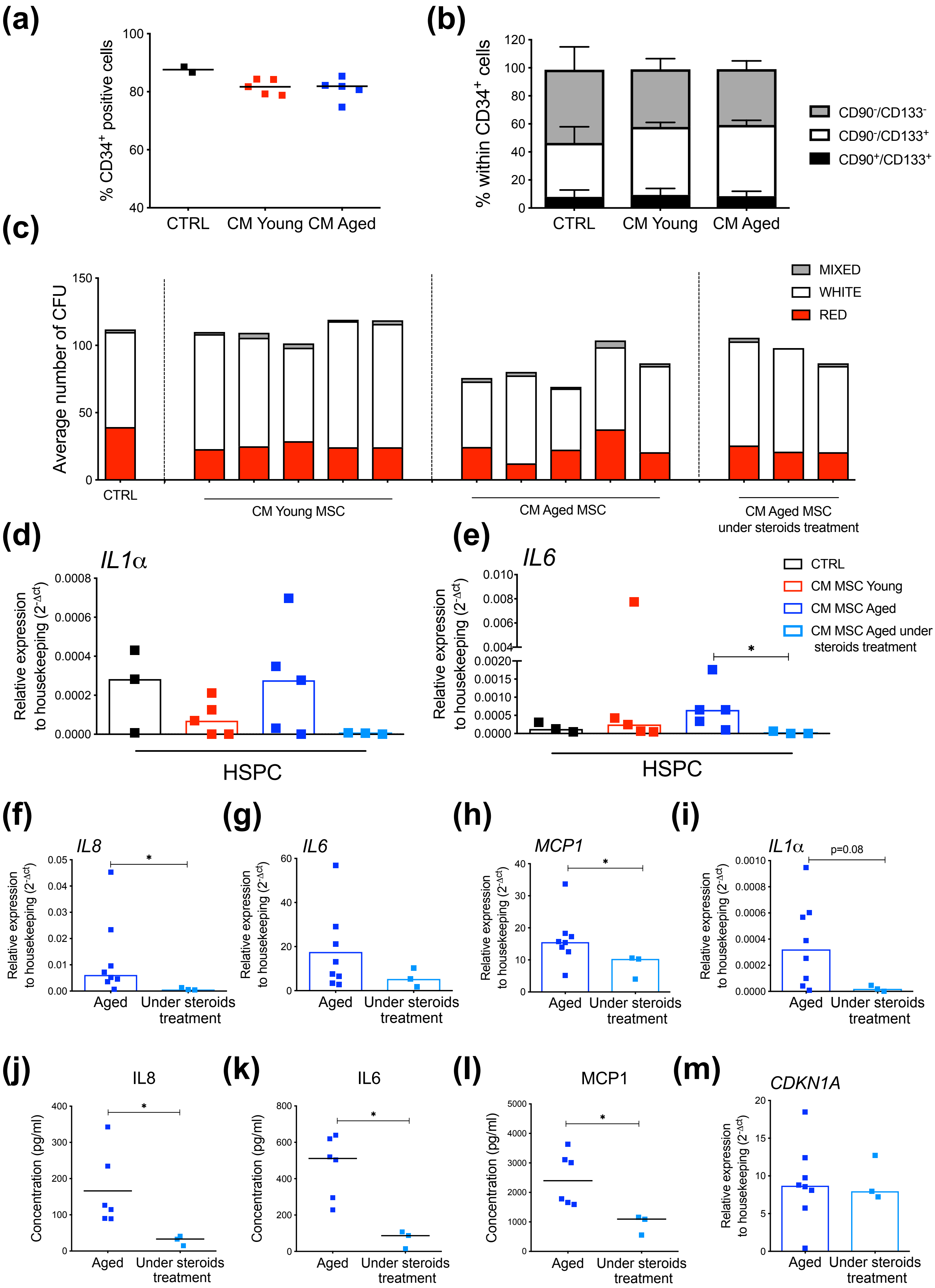

Supplement: Supplementary file 1 [file ACEL-18-e12933-s001.pdf]
