# Supplementary material for: An early‐senescence state in aged mesenchymal stromal cells contributes to hematopoietic stem and progenitor cell clonogenic impairment through the activation of a pro‐inflammatory program
Source: Aging Cell. 2019 Mar 3;18(3):e12933. doi: 10.1111/acel.12933 (PMC6516180; doi:10.1111/acel.12933)
Supplement: Supplementary file 2 [file ACEL-18-e12933-s002.pdf]

Supplementary Figure Legends

Figure S1: Differentiation ability of young and aged MSC.

(a-c) Gene expression levels of *PPARγ*, *FABP4* and *LPL* adipogenic genes at steady state in young and aged MSC (young, n=4; aged, n=8). *p*-value was determined by Mann-Whitney test; $*p < 0.05$. **(d-f)** Quantitative Real time PCR for *PPARγ, FABP4* and *LPL* adipogenic genes in young and aged MSC at basal level and post-differentiation with adipogenic medium for 21 days. Gene expression data are represented as $2^{-\Delta CT}$ relative to *ACTNB* housekeeping. Histograms indicate median $2^{-\Delta CT}$ values (young, n=3; aged, n=4). *p*-value was determined by Mann-Whitney test; $*p < 0.05$. **(g)** Representative pictures of differentiated young and aged MSC stained with Oil Red O; Red signal indicates lipid droplets. **(h-j)** Gene expression levels of *RUNX2*, *SPARC* and *COL1A2* osteogenic genes at steady state in young and aged MSC (young, n=4; aged, n=8). **(k-m)** Quantitative Real time PCR for early *RUNX2, SPARC* and *COL1A2* osteogenic genes in young and aged MSC at basal level and post-differentiation with osteogenic medium for 21 days. Gene expression data are represented as $2^{-\Delta CT}$ relative to *ACTNB*. Bars indicate median $2^{-\Delta CT}$ values (young, n=3; aged, n=3). *p*-value was determined by Mann-Whitney test; $*p < 0.05$. **(n)** Representative pictures of differentiated young and aged MSC stained with Alizarin Red. In all panels, each squared dot represents MSC from an individual donor (red=young; blue=aged).

Figure S2: Analysis of lipofuscin, ROS levels, oxidized DNA and DDR markers in young and aged MSC.

(a) Representative picture for SenTraGor staining in young (n=4; Y1-4) and aged (n=6; A1-6) MSC. White arrows indicate positive SenTraGor Staining. Nuclei were counterstained with DAPI. Senescent BJ fibroblasts were used as a positive control while young MSC (Y3) stained in the absence of anti-biotin antibody or SenTraGor were used as negative controls. Scale bar= 20µm. **(b)** ROS levels as measured by CellROX staining in young and aged MSC. Lines indicate mean values ± SEM. ROS levels were calculated as DMFI relative to unstained controls (young, n=4; aged, n=4); *p*-value was determined by Mann-Whitney test; $*p < 0.05$. **(c)** Relative mRNA expression of *FOXO4* measured by quantitative Real-Time PCR. Gene expression data are represented as $2^{-\Delta CT}$ relative to *GUSB* as scatter dot plots, bars indicate median $2^{-\Delta CT}$ values (young, n=6; aged, n=8); *p*-value was determined by Mann-Whitney test; $*p < 0.05$. **(d)** FACS analysis for 8-oxo-dG levels in young and aged MSC expressed as percentage of positive cells and represented as scatter dot plots (young, n=4; aged, n=7). Each squared dot represents MSC from an individual donor (red=young; blue=aged); **(e-f)** Representative confocal images for pATM (pATMS1981) and γH2A.X in young and aged MSC at early passages. Human BJ cells induced into senescence by irradiation (20Gy)

were used as positive control for DDR foci staining. For senescent cells numbers indicate the percentage of DDR-positive cells. For young and aged MSC numbers indicate the median of the percentage of foci positive cells for γH2A.X (3 young and 4 aged MSC) and for pATM staining (3 young and 3 aged). Nuclei were counterstained with DAPI. Scale bar=20 μm.

Figure S3: Aged MSC display reduced immunomodulatory properties.

(a) Schematic representation of the *in vitro* model to assess MSC immune-modulatory properties in suppressing PBMC proliferation. **(b)** *In vitro* immunomodulatory effect of young and aged MSC on human healthy donor peripheral blood mononuclear cells (PBMC). The graph shows the percentage of residual proliferation of PHA-stimulated PBMC in presence of young and aged MSC at different ratios (1:2, 1:20 and 1:200), calculated by measuring ^{3}H-thymidine incorporation after 72 hours of co-culture. Dash line represents PBMC proliferation in the absence of MSC considered as 100% and used to normalize PBMC proliferation in presence of young or aged MSC (young, n=3; aged, n=4). Each squared dot represents MSC from an individual donor (red=young; blue=aged). *p*-value was determined by Mann-Whitney test; $*p < 0.05$. **(c-f)** Gene expression analysis for *CXCL10, PTGS2, TGF-β1* and *IDO1* by quantitative Real Time PCR in young and aged MSC at early passages in culture. Gene expression data are represented as $2^{-\Delta CT}$ relative to housekeeping genes in scatter dot plots, bars indicate median $2^{-\Delta CT}$ values (young, n=4; aged, n=8); *p*-values was determined by Mann-Whitney test; $*p < 0.05$.

Figure S4: SASP activation in human bone marrow samples and MSC aging.

(a-f) Plasma levels of IL6, IL8, MCP1, IL1α, TNFα, and IL1β in human bone marrow samples from young and aged donors measured by Luminex technology (young, n=6; aged, n=8); Values of individual donors are represented as scatter dot plot, lines indicate median values. *p*-value was determined by Mann-Whitney test; $**p < 0.01$; $*p < 0.05$. **(g-i)** Relative mRNA expression of *IL6, MCP1* and *IL1β* late passages young (n=3) and aged MSC (n=3). Gene expression data are represented as $2^{-\Delta CT}$ relative to *GUSB* as scatter dot plots, histograms indicate median $2^{-\Delta CT}$ values. *p*-value was determined by Mann-Whitney test; $*p < 0.05$. **(j-m)** Relative mRNA expression of HSPC supportive genes *CXCL12*, *VCAM, VEGF* and *ANGPT1* measured by quantitative Real-Time PCR in young (n=6) and aged (n=8) MSC. Gene expression data are represented as $2^{-\Delta CT}$ relative to *GUSB* as scatter dot plots, bars indicate median $2^{-\Delta CT}$ values.

Figure S5: Paracrine effects of CM from aged MSC of subjects under chronic steroids treatment on HSPC functionality.

(a) Percentage of $CD34^+$ HSPC measured by FACS after 4 days in culture with 1:1 StemSpan/CM derived from young or aged MSC. $CD34^+$ cells grown without CM in StemSpan were used as control. **(b)** Histograms represent the mean percentage ± SD of primitive ($CD90^+/CD133^+$), early ($CD90^+/CD133^-$) and committed ($CD90^-/CD133^-$) progenitors within total $CD34^+$ cells for each donor analyzed at 96 hours; (CM from young, n=4; aged, n=4). HSPC from two independent CB-derived donors were employed. (**c**) Number of HSPC colonies in methylcellulose analyzed at 96 hours post exposure to CM derived from healthy young, aged MSC or MSC derived from aged donors under steroids treatment. Red, white and light grey bars represent erythroid, myeloid and mixed colonies, respectively. $CD34^+$ cells grown without CM (CTRL) were used as control. (**d-e**) Gene expression analysis of *IL1α* and *IL6* by quantitative Real Time PCR in CB-derived $CD34^+$ cells cultured for 96 hours either in absence (CTRL) or in presence of CM derived from young MSC, aged MSC and aged MSC under steroids treatment. Gene expression data are represented as $2^{-\Delta CT}$ relative to *GUSB* housekeeping, histograms indicate median $2^{-\Delta CT}$ values ($CD34^+$ CTRL n=3; young, n=5; aged, n=5; aged under steroids treatment, n=3). *p*-value was determined by Mann-Whitney test; $*p < 0.05$. (**f-i**) Relative mRNA expression of *IL8, IL6, MCP1* and *IL1α* in healthy aged or in MSC derived from aged donors under steroids treatment (aged, n=8, aged under steroids treatment, n=3). Gene expression data are represented as $2^{-\Delta CT}$ relative to *GUSB* as scatter dot plots, histograms indicate median $2^{-\Delta CT}$ values. *p*-values was determined by Mann-Whitney test; $*p < 0.05$. **(j-l)** Levels of IL8, IL6 and MCP1 measured by Luminex assay in CM collected from early passages aged MSC and aged MSC of subjects under steroids treatment. Values are represented as scatter dot plot, lines indicate median values; (aged, n=6; aged under steroids treatment, n=3). *p*-value was determined by Mann-Whitney test; $*p < 0.05$. (**m**) Relative mRNA expression of *CDKN1A* in healthy aged or in MSC derived from aged donors under steroids treatment (aged, n=8, aged under steroids treatment, n=3). Gene expression data are represented as $2^{-\Delta CT}$ relative to *GUSB* as scatter dot plots, bars indicate median $2^{-\Delta CT}$ values.

Supplementary Table 1.

List of pediatric, young and aged MSC samples employed in each figure panel.

	Number of donors		
Figures	Pediatric MSC	Young Adults MSC	Aged MSC
1b (day7, day14)	4	6	12; 7
1d-e	2	4	8
1g-h	2	4	8
2c	2	2	4
2d-f	1	-	2
2g	3	3	8
2h	3	3	8
2j	3	1	8
3b	3	2	5
3d	3	2	5
3f	3	2	5
3g	3	4	7
3i	2	1	3
3j	2	1	3
4a	2	4	8
4b-d	3	3	8
4e	2	4	8
4f-k	2	3	6
5b-e	2	3	5
6a-c	-	-	3
6d-f	-	-	3
S1a-c	-	4	8
S1d-f	-	3	4
S1h-j	-	4	8
S1k-m	-	3	3
S2a	2	2	6
S2b	3	1	4
S2c	3	3	8
S2d	2	2	7
S2e	2	2	4
S2f	2	1	3
S3b	1	2	4
S3c	2	2	8
S3d-f	-	4	8
S4a-f	-	6	8
S4g-i	2	1	3
S4j-m	3	3	8
S5a-b	2	2	5
S5c-e	2	3	5
S5f-i	-	-	8
S5j-l	-	-	6
S5m	-	-	8

Supplementary Table 2

Sequences of RT-qPCR primers employed.

	Forward	*Reverse*
ACTNB	ACAGAGCCTCGCCTTTGC	ACTCCATGCCCAGGAAGGAA
PPARG	TCAGAAATGCCTTGCAGTGG	TATCACTGGAGATCTCCGCCAA
LPL	CCGCCGACCAAAGAAGAGAT	TAGCCACGGACTCTGCTACT
FABP4	AAACTGGTGGTGGAATGCGT	GCGAACTTCAGTCCAGGTCA
RUNX2	CCGGAATGCCTCTGCTGTTA	TGTCTGTGCCTTCTGGGTTC
SPARC	ATTGACGGGTACCTCTCCCA	GAAAAAGCGGGTGGTGCAAT
COL1A2	ACAAGGCATTCGTGGCGATA	ACCATGGTGACCAGCGATAC
TGF-β1	CGATGAGCAGCTTTTCCAGA	AGTGAACCCGTTGATGTCCA
IDO1	TGGCCAGCTTCGAGAAAGAG	TGGCAAGACCTTACGGACATC
PTGS2	CAAATTGCTGGCAGGGTTGC	AGGGCTTCAGCATAAAGCGT
IL8	CATCTCACTGTGTGTAAACATGAC	CCTTGGCAAAACTGCACCTTCAC
IL1β	GCTCAAGTGTCTGAAGCAGCC	CAGCTTCAAAGAACAAGTCATCCT
IL6	GATTCAATGAGGAGACTTGCCTGG	CTCACTACTCTCAAATCTGTTCTGG
IL1α	GGTTGAGTTTAAGCCAATCCA	TGCTGACCTAGGCTTGATGA
MCP1	CTGTGATCTTCAAGACCATTGTG	AGTTTGGGTTTGCTTGTCCAG
CDKN2A	CCAACGCACCGAATAGTTACG	GCGCTGCCCATCATCATG
CDKN1A	CAGCATGACAGATTTCTACCACTC	CTCGCGCTTCCAGGACTG
GUSB	CTGACACCTCCAAGTATCCCAAG	GTCGTGTACAGAAGTACAGACCGC
FOXO4	GAGCCAGATCCCTGAGTCATG	GGTAGCTCTAAAGCAGGGTCC
VEGFA	CATCTTCAAGCCATCCTGTG	GGAAGCTCATCTCTCCTATG
CXCL12	TGCCCTTCAGATTGTAGCCC	CGAGTGGGTCTAGCGGAAAG
VCAM1	GACCTTCATCCCTACCATTG	TTCACAGAACTGCCTTCCTCCA
ANGPT1	ACATGGGCAATGTGCCTACA	TCTCAAGTTTTTGCAGCCACTG
CXCL10	GCAGAGGAACCTCCAGTCTCAGC	GAGAGAGGTACTCCTTGAATGC
